# Investigation of the mechanisms of VEGF-mediated compensatory lung growth: the role of the VEGF heparin-binding domain

**DOI:** 10.1038/s41598-021-91127-0

**Published:** 2021-06-04

**Authors:** Lumeng J. Yu, Victoria H. Ko, Duy T. Dao, Jordan D. Secor, Amy Pan, Bennet S. Cho, Paul D. Mitchell, Hiroko Kishikawa, Diane R. Bielenberg, Mark Puder

**Affiliations:** 1 Vascular Biology Program, Boston Children’s Hospital, Harvard Medical School, Boston, MA 02115 USA; 2 Department of Surgery, Boston Children’s Hospital, Harvard Medical School, 300 Longwood Ave, Fegan 3, Boston, MA 02115 USA; 3 Institutional Centers for Clinical and Translational Research, Boston Children’s Hospital, Boston, MA 02115 USA

**Keywords:** Respiratory tract diseases, Paediatric research, Preclinical research, Experimental models of disease, Translational research, Drug development

## Abstract

Morbidity and mortality for neonates with congenital diaphragmatic hernia-associated pulmonary hypoplasia remains high. These patients may be deficient in vascular endothelial growth factor (VEGF). Our lab previously established that exogenous VEGF164 accelerates compensatory lung growth (CLG) after left pneumonectomy in a murine model. We aimed to further investigate VEGF-mediated CLG by examining the role of the heparin-binding domain (HBD). Eight-week-old, male, C57BL/6J mice underwent left pneumonectomy, followed by post-operative and daily intraperitoneal injections of equimolar VEGF164 or VEGF120, which lacks the HBD. Isovolumetric saline was used as a control. VEGF164 significantly increased lung volume, total lung capacity, and alveolarization, while VEGF120 did not. Treadmill exercise tolerance testing (TETT) demonstrated improved functional outcomes post-pneumonectomy with VEGF164 treatment. In lung protein analysis, VEGF treatment modulated downstream angiogenic signaling. Activation of epithelial growth factor receptor and pulmonary cell proliferation was also upregulated. Human microvascular lung endothelial cells (HMVEC-L) treated with VEGF demonstrated decreased potency of VEGFR2 activation with VEGF121 treatment compared to VEGF165 treatment. Taken together, these data indicate that the VEGF HBD contributes to angiogenic and proliferative signaling, is required for accelerated compensatory lung growth, and improves functional outcomes in a murine CLG model.

## Introduction

Despite significant advances in surgical and intensive care management over the past three decades, morbidity and mortality for neonates with congenital diaphragmatic hernia (CDH)-associated pulmonary hypoplasia (PH) remains high. Patients with CDH and severe PH may require cardiopulmonary support with extracorporeal membrane oxygenation (ECMO). Mortality for patients with CDH requiring ECMO approaches 50%^1,2^. Animal models and human patients with CDH-associated PH may be deficient in vascular endothelial growth factor (VEGF), especially in the alveolar stage of lung development^3,4^. During lung development, mesenchymal and alveolar epithelial cells secrete VEGF, which drives endothelial cell proliferation and results in coordinated bronchovascular growth^5–7^. As such, increasing pulmonary VEGF during lung development, particularly during a critical period of alveolarization, much of which occurs postnatally in humans, has become an attractive therapeutic target for developmental lung diseases including CDH-associated PH, bronchopulmonary dysplasia, and respiratory distress syndrome^3,8–10^.

Murine left pneumonectomy-induced compensatory lung growth (CLG) shares molecular patterning similar to developmental alveolarization and serves as a model to study pulmonary hypoplastic diseases^11,12^. CLG is particularly reflective of the post-natal alveolar stage of human lung development, as it is characterized by expansion of the remaining lung to the full volume of both lungs via septal surface expansion and re-alveolarization^12^. Our laboratory has previously demonstrated that CLG in mice completes at post-operative day (POD) eight to ten^13^. In accordance with the prior observation of decreased VEGF mRNA expression in patients with CDH in the alveolar stage of development, our group administered exogenous VEGF164, the most abundant murine isoform of the protein, in a murine CLG model, and demonstrated accelerated CLG to completion by POD4^13,14^. However, VEGF164 contains a heparin-binding domain (HBD), which interacts with heparin. Prior studies have revealed that exogenous heparin impairs VEGF-mediated accelerated CLG^15^. As heparin is the most commonly used anticoagulant, especially in patients requiring ECMO, the potentially deleterious interaction between VEGF and heparin is of particular concern.

In working towards the goal of translating systemic VEGF therapy into clinical practice for PH, we hypothesized that a VEGF protein lacking heparin-binding ability may potentially be effective in accelerating CLG without interacting with exogenous heparin given clinically. The VEGF-A pre-mRNA is alternatively spliced to yield three major protein isoforms in mice and humans, VEGF120/164/188 and VEGF121/165/189, respectively. In particular, VEGF120/121, the smallest of the major isoforms, lacks exons 6 and 7, which encode the HBD^16^. The complete absence of heparin-binding VEGF isoforms 164/188 in a transgenic mouse model resulted in non-viable pups with completed pulmonary development but pruned lung vasculature, indicating that VEGF120 alone is not sufficient to achieve mature pulmonary function^17^. However, delivery of exogenous VEGF120 in the presence of normal, endogenous VEGF produces a similar pro-angiogenic response compared to provision of HBD-containing exogenous VEGF^18^. To our knowledge, no prior studies have assessed the efficacy of VEGF120 in the murine CLG model, or evaluated the potential for VEGF to affect functional outcomes such as exercise tolerance. The purpose of our current study was to assess the efficacy of exogenous VEGF120 in accelerating CLG in a model of murine left pneumonectomy. We also aimed to further delineate the role of the HBD in VEGF-mediated pulmonary growth, development, and function.

## Results

### VEGF treatment increases compensatory lung growth in a dose-dependent manner

**Figure 1:**
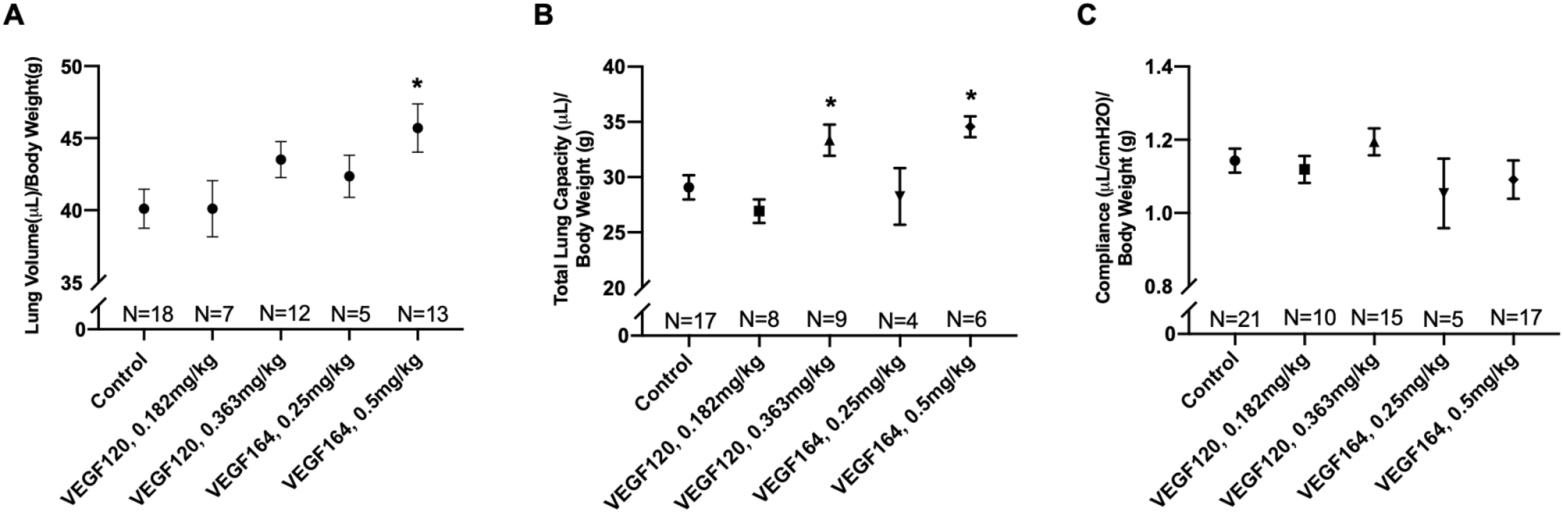
Lung volume and pulmonary function testing. Both VEGF164 and VEGF120 increased lung volume, as measured by the water displacement method (**A**), and total lung capacity on pulmonary function testing (**B**). However, only the higher dose of VEGF164 induced a significant increase in lung volume, while both the higher dose of VEGF164 and the the equimolar dose of VEGF120 significantly increased total lung capacity. Compliance was not significantly different between VEGF-treated mice and controls (**C**). Statistical analysis of lung volume, total lung capacity, and compliance was performed by ANOVA with Holm-Sidak correction for multiple comparisons. Results are expressed as mean ± SE. *P < 0.05. Mouse cohorts of pneumonectomies were performed in three independent repeats. On any given operative day, a control group of mice was included.

Mice treated with VEGF164 demonstrated a dose-dependent increase in lung volume normalized to body weight compared to saline-treated control mice. While the lower dose of 0.25 mg/kg VEGF164 mildly increased lung volume compared to control, only the higher, previously validated dose of 0.5 mg/kg produced a statistically significant increase (45.7 ± 1.7 vs. 40.1 ± 1.3 μL/g, *P* = 0.02) (Fig. 1A). Similarly, VEGF120 demonstrated a milder dose-dependent increase in lung volume, although neither dose reached statistical significance compared to saline control. Both the higher doses (0.0256 mmol/kg), 0.5 mg/kg VEGF164 (34.6 ± 0.94 vs. 29.1 ± 1.1 μL/g, *P* = 0.03) and equimolar 0.363 mg/kg VEGF120 (33.4 ± 1.4 vs. 29.1 ± 1.1 μL/g, *P* = 0.04), significantly increased total lung capacity (TLC) compared to control (Fig. 1B). However, neither of the lower doses impacted TLC. There were no significant differences in compliance among treatment groups (Fig. 1C). The increase in lung volume by water displacement and TLC was unique to active VEGF treatment. Inactive VEGF120 or VEGF164 did not produce a significant increase in lung volume or TLC at POD4 after pneumonectomy (Supplemental Fig. S1A,B). In addition, VEGF treatment only affected the regenerating lung, but not organs that were not regenerating such as the liver, kidney, or spleen. Non-regenerative organs had comparable weights on POD4 of VEGF120 or VEGF164 treatment compared to control treatment (Supplemental Fig. S2).

### VEGF164 improves alveolarization on morphometric analysis

**Figure 2:**
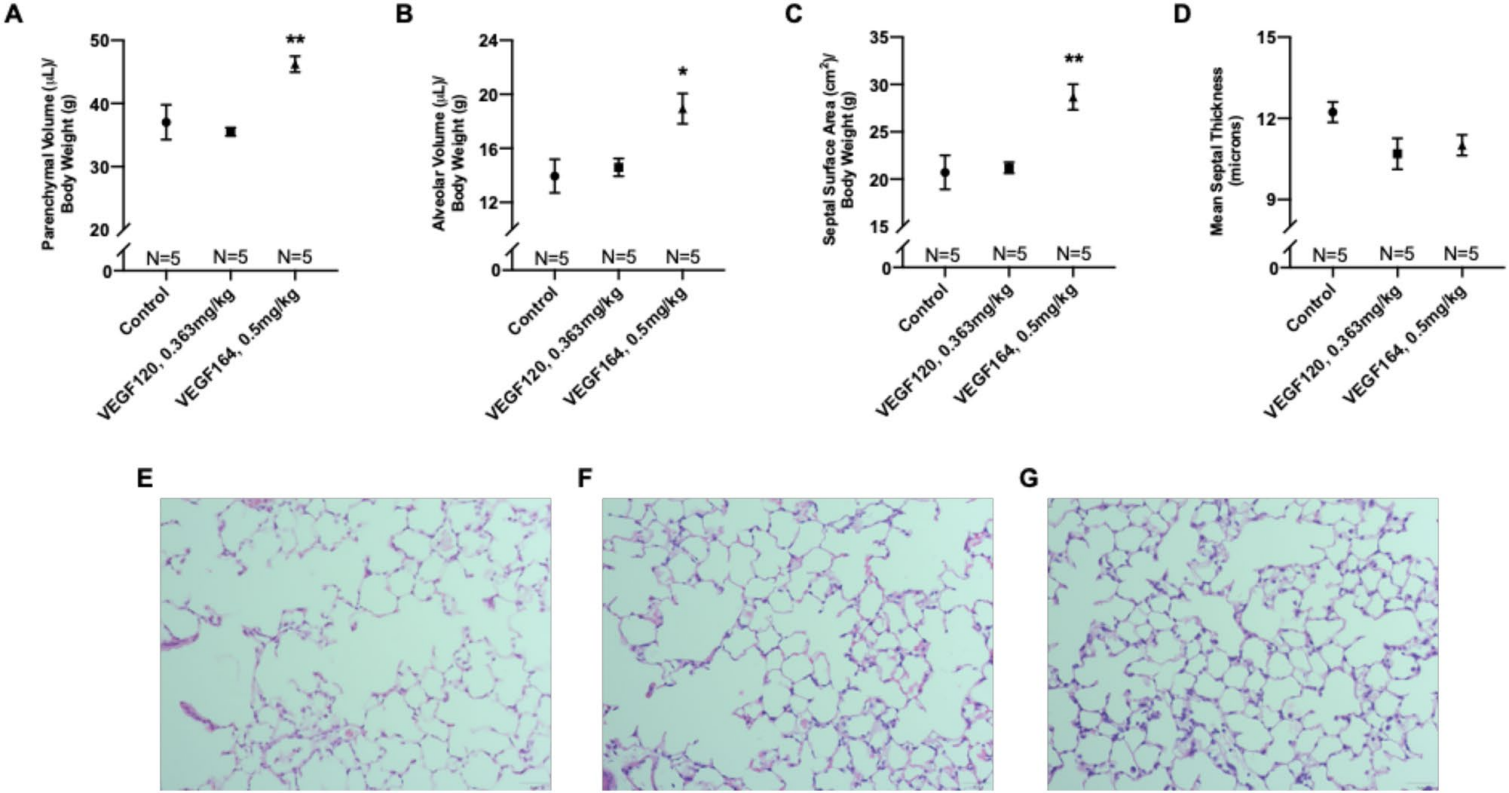
Morphometric analysis of lung tissue. VEGF164-treated mice exhibited significant increases in parenchymal volume (**A**), alveolar volume (**B**), and septal surface area (**C**), while VEGF120 did not. VEGF treatment also decreased mean septal thickness, although this did not reach statistical significance (D). Representative micrographs at × 200 magnification of hematoxylin and eosin-stained, control (**E**), VEGF120 (**F**), and VEGF164 (**G**) lung sections demonstrate increased alveolarization with VEGF164 treatment (**G**). Statistical analysis of morphometrics was performed by ANOVA with Holm-Sidak correction for multiple comparisons. Results are expressed as mean ± SE. *P < 0.05. Results are expressed as mean ± SE. *P < 0.05, **P < 0.01.

Compared to controls (representative histology in Fig. 2E), VEGF164 significantly increased parenchymal volume (46.2 ± 1.3 vs. 37.0 ± 2.8 μL/g, *P* = 0.007), alveolar volume (18.9 ± 1.1 vs. 14.0 ± 1.2 μL/g, *P* = 0.01), and septal surface area (28.7 ± 1.4 vs. 20.7 ± 1.8 cm^2^/g, *P* = 0.003) normalized for body weight (Fig. 2A–C,G). Meanwhile, VEGF120 did not affect alveolarization (Fig. 2A–C,F). Decreased mean septal thickness, which may facilitate ease of gas exchange, was observed in both VEGF120- and VEGF164-treated lungs, although this did not reach statistical significance (Fig. 2D).

### VEGF164 improves exercise tolerance after left pneumonectomy

**Figure 3:**
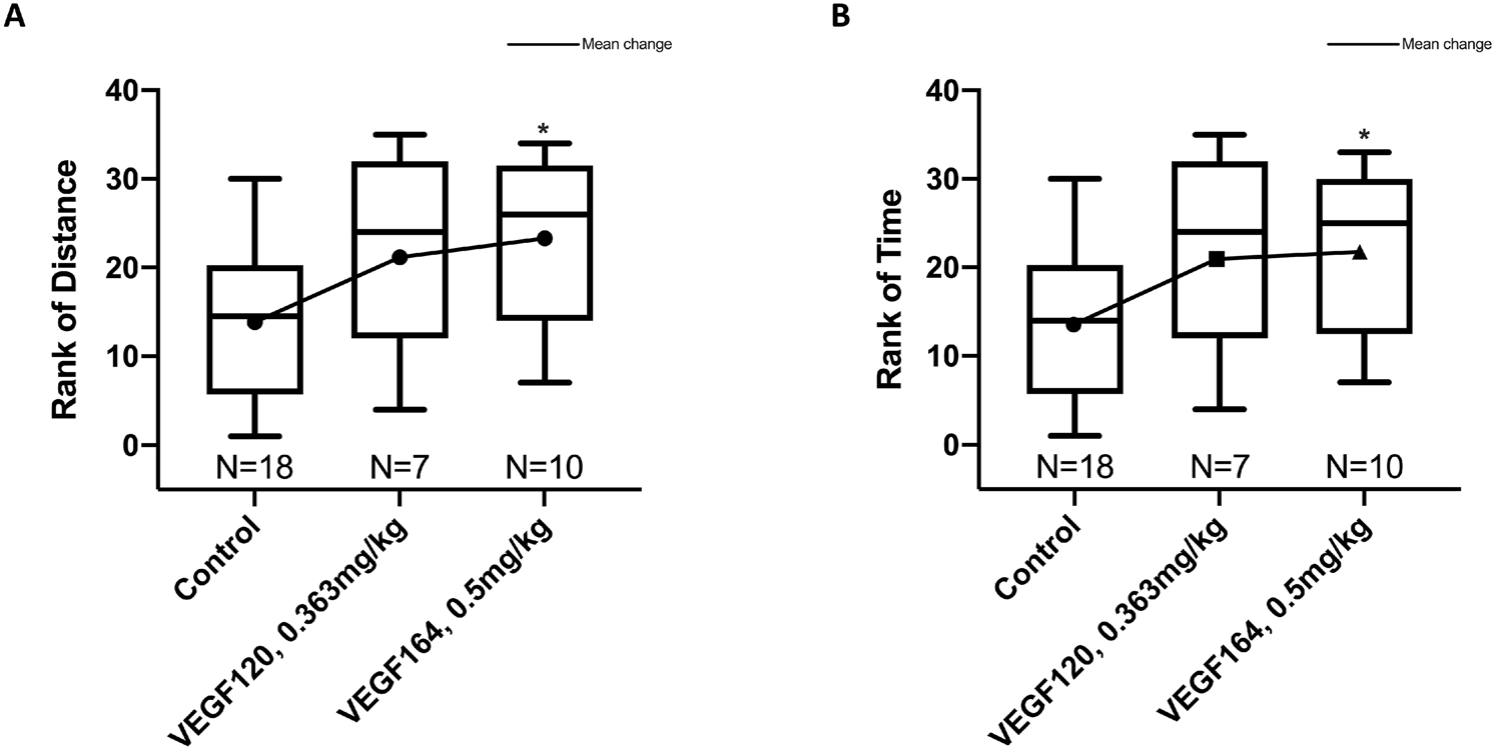
Treadmill exercise tolerance testing. Rank-based analysis of covariance, adjusted for baseline outcome and multiple pairwise comparisons, showed greater mean change in VEGF164 vs. control (*P* = 0.03 for distance and for time), with no differences between VEGF120 and control (*P* = 0.11 for distance and *P* = 0.09 for time) and no differences between VEGF120 and VEGF164 (*P* = 0.64 and *P* = 0.66 for distance and time, respectively) (**A**, **B**). Statistical analysis of exercise tolerance was performed by nonparametric ANCOVA of the change in distance or time (POD4 minus baseline) and adjusted for multiple pairwise corrections using the Holm-Sidak correction. Results are expressed as median and interquartile range (IQR), with mean values. *P < 0.05. Mouse cohorts of pneumonectomies were performed in three independent repeats. On any given operative day, a control group of mice was included.

Treadmill exercise tolerance testing (TETT) was used to assess functional outcomes with VEGF treatment after murine left pneumonectomy, as it may be representative of functional pulmonary outcomes in human patients. Mice were acclimated to the treadmill and underwent TETT two days prior to surgery to obtain a baseline, and again on POD4 to assess treatment effect. Distance run and time spent running on POD4 were normalized to differences in baseline values on each mouse to account for any intrinsic physiologic or behavioral differences between mice. When comparing distance run and time spent running on the treadmill post-surgery and treatment to pre-operative, VEGF164-treated mice exhibited improved exercise tolerance after left pneumonectomy compared to control mice [median 5.6 (IQR 0.9, 13.6) vs. − 0.4 (− 7.6, 2.4) minutes and 77 (11, 204) vs. − 5 (− 106, 18) meters, respectively)]. These differences were maintained after adjusting for pre-operative baseline and multiple pairwise comparisons (*P* = 0.03 by rank-based analysis of covariance; Fig. 3). Although VEGF120-treated mice had increased exercise distance and time (median 58 (− 27, 275) meters and 4.2 (− 2.0, 18.3) minutes, respectively), the change was not statistically different from either control- (*P* = 0.11 for distance; *P* = 0.09 for time) or VEGF164-treated mice (*P* = 0.64 for distance; *P* = 0.66 for time). Additionally, VEGF120 or VEGF164 treatment did not induce a significant increase in hematocrit (Supplemental Fig. S1C) or endothelial cell proliferation of non-regenerative tissues (particularly skeletal muscle) (Supplemental Fig. S3), to alternatively account for improvement in exercise tolerance.

### Exogenous VEGF stimulates downstream VEGF and paracrine signaling

**Figure 4:**
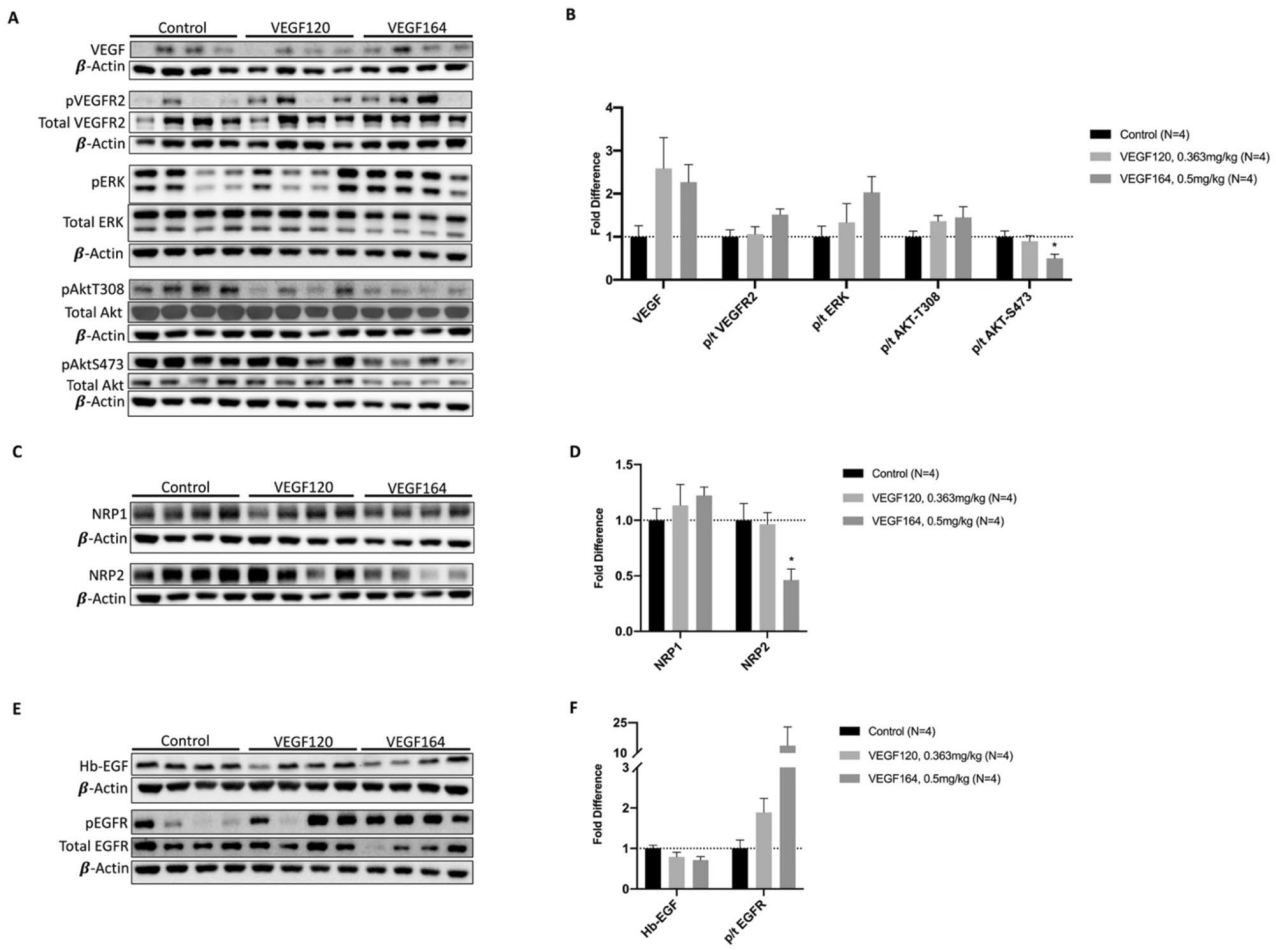
Lung tissue protein analysis. VEGF treatment with either VEGF120 or VEGF164 increased lung tissue expression of VEGF ligand, activated VEGFR2 (phosphorylated/total receptor), and downstream proliferation markers activated ERK (phosphorylated/total ERK), and activated AKT-T308 (**A**, **B**) [Full-length uncropped blots are provided in Supplementary Information File 1.] However, activated AKT-S473 was significantly downregulated. VEGF treatment also significantly downregulated co-receptor neuropilin-2 (NRP2), while co-receptor neuropilin-1 (NRP1) expression was not significantly different between groups (**C**, **D**). [Full-length uncropped blots are provided in Supplementary Information File 2.] While heparin-binding epithelial growth factor (Hb-EGF) expression was unchanged with treatment, activated epithelial growth factor receptor (phosphorylated/total EGF receptor) was upregulated, indicating potential paracrine signaling by VEGF treatment (**E**, **F**). [Full-length uncropped blots are provided in Supplementary Information File 3.] Antibodies probed on the same blot were incubated on cut membranes to facilitate specificity of binding and ability to probe multiple antibodies at one time. The β-actin loading control for each membrane is displayed below the antibodies probed on the same membrane. For membranes probed with phosphorylated and total protein, total protein antibody was used stripping the membrane as appropriate. Full length uncropped membranes are provided in the Supplementary Information Files. Statistical analysis of protein expression was performed by ANOVA with Holm-Sidak correction for multiple comparisons. Results are expressed as mean ± SE. *P < 0.05. Each lane represents lung protein lysate from a different mouse. Western blots were performed one to three times for quality control, and a representative image is shown.

Protein analysis of lung tissue was performed to characterize the effects of exogenous VEGF on pulmonary cell signaling. Both VEGF120 and VEGF164 increased pulmonary levels of VEGF ligand, although this did not reach statistical significance, in part due to differences among biological replicates (Fig. 4A,B). Similarly, there was a nonsignificant increase in activation of VEGF receptor 2 (p-Y1175t VEGFR2) with provision of exogenous VEGF compared to control. Downstream effectors of VEGF signaling, including the MAPK/ERK and AKT pathways, were also increased, as demonstrated by increased phosphorylated to total p44/42 (ERK) and phosphorylated to total AKT-T308. However, activated AKT-S473 (p/t AKT-S473) was significantly downregulated in the VEGF164-treated group compared to control (0.50 ± 0.10 vs. 1.0 ± 0.14-fold, *P* = 0.03) (Fig. 4A,B).

Neuropilins (NRPs) are neuronal receptors for class 3 semaphorins, but also act as VEGF co-receptors to facilitate angiogenesis^19^. Provision of either VEGF120 or VEGF164 only slightly increased NRP1 expression. However, VEGF164 treatment significantly reduced NRP2 expression compared to control (0.46 ± 0.10 vs. 1.0 ± 0.15-fold, *P* = 0.02) (Fig. 4C,D).

Heparin-binding epidermal growth factor-like factor (Hb-EGF) levels were mostly unchanged between VEGF-treated lung tissue and controls; however, EGFR activation, as measured by phosphorylated to total EGFR, was increased in VEGF-treated groups, although not reaching statistical significance (Fig. 4E,F).

### VEGF treatment induces greater increases in total lung cell proliferation than lung endothelial cell proliferation

**Figure 5:**
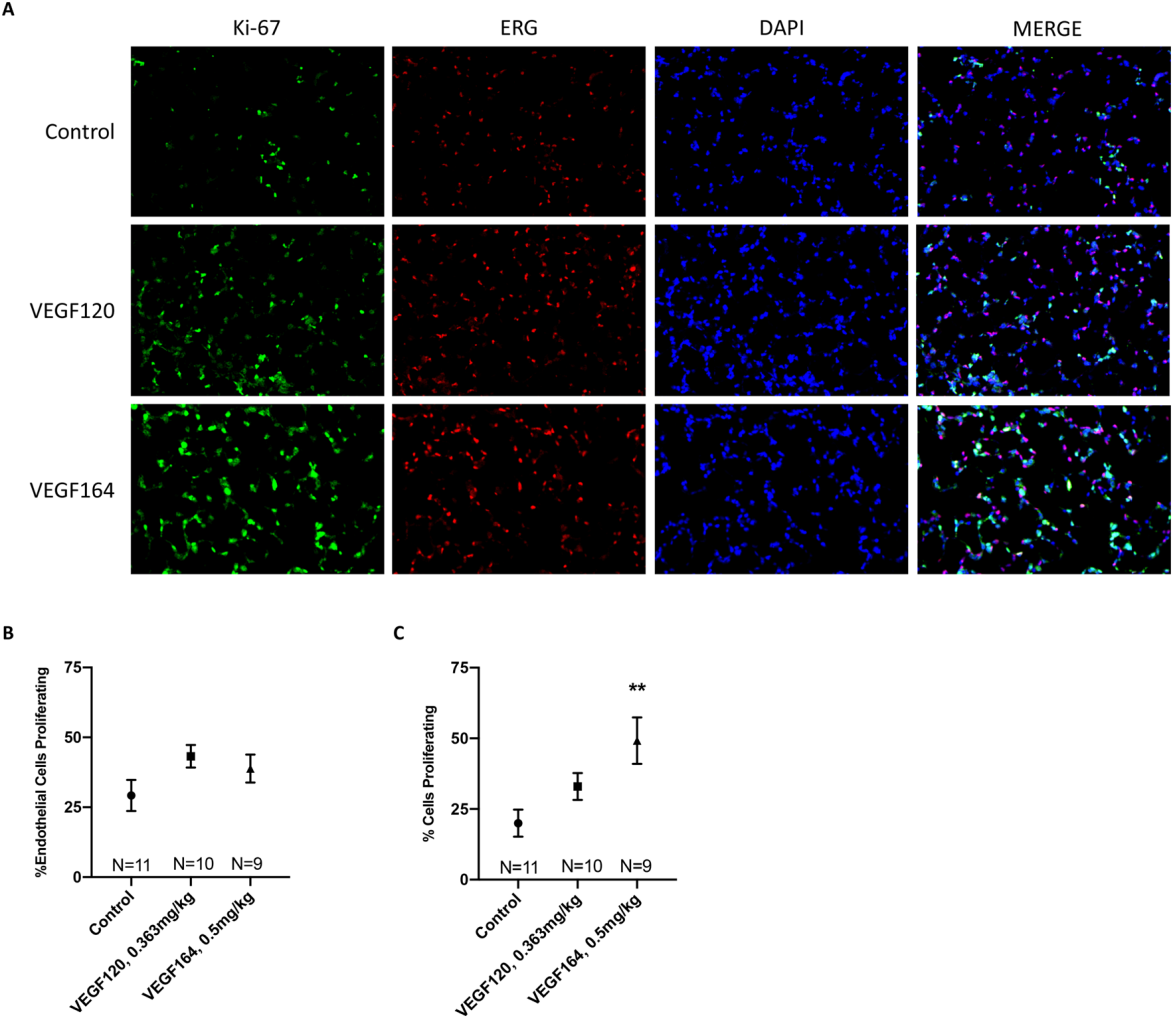
Immunohistochemistry. Representative micrographs at × 200 magnification of immunofluorescence-stained lung tissue with Ki-67 (green) and ERG (red) with nuclear DAPI (blue) demonstrated increased endothelial cell proliferation and significantly increased global pulmonary proliferation with VEGF164 treatment, which is more pronounced than VEGF120 treatment, compared to control (**A**). Quantification of co-staining indicated more substantial increases of total cell proliferation in the lung (Ki-67/DAPI) (**C**) compared to endothelial cell proliferation (Ki-67/ERG/DAPI) (**B**), which did not reach statistical significance. Statistical analysis of immunofluorescence co-staining quantification was performed by ANOVA with Holm-Sidak correction for multiple comparisons. Results are expressed as mean ± SE. *P < 0.05. Results are expressed as mean ± SE. **P < 0.01.

Immunohistochemistry (IHC) was performed to assess endothelial and epithelial cell proliferation in VEGF-treated lung tissue (Fig. 5A). Compared to controls, both VEGF120 and VEGF164 increased endothelial cell proliferation, as demonstrated by co-staining of Ki-67, a proliferative marker, and ERG, a nuclear endothelial cell marker^20^, although not reaching statistical significance (Fig. 5B). However, when quantitating the percentage of total proliferating pulmonary cells (including both endothelial and epithelial cells), VEGF164 induced a significant increase in global pulmonary cell proliferation (endothelial and epithelial cells) compared to controls (49.2 ± 8.2 vs. 20.0 ± 4.8 percent, *P* = 0.004), while VEGF120 did not (Fig. 5C).

### The VEGF heparin-binding domain increases VEGFR2 activation

**Figure 6:**
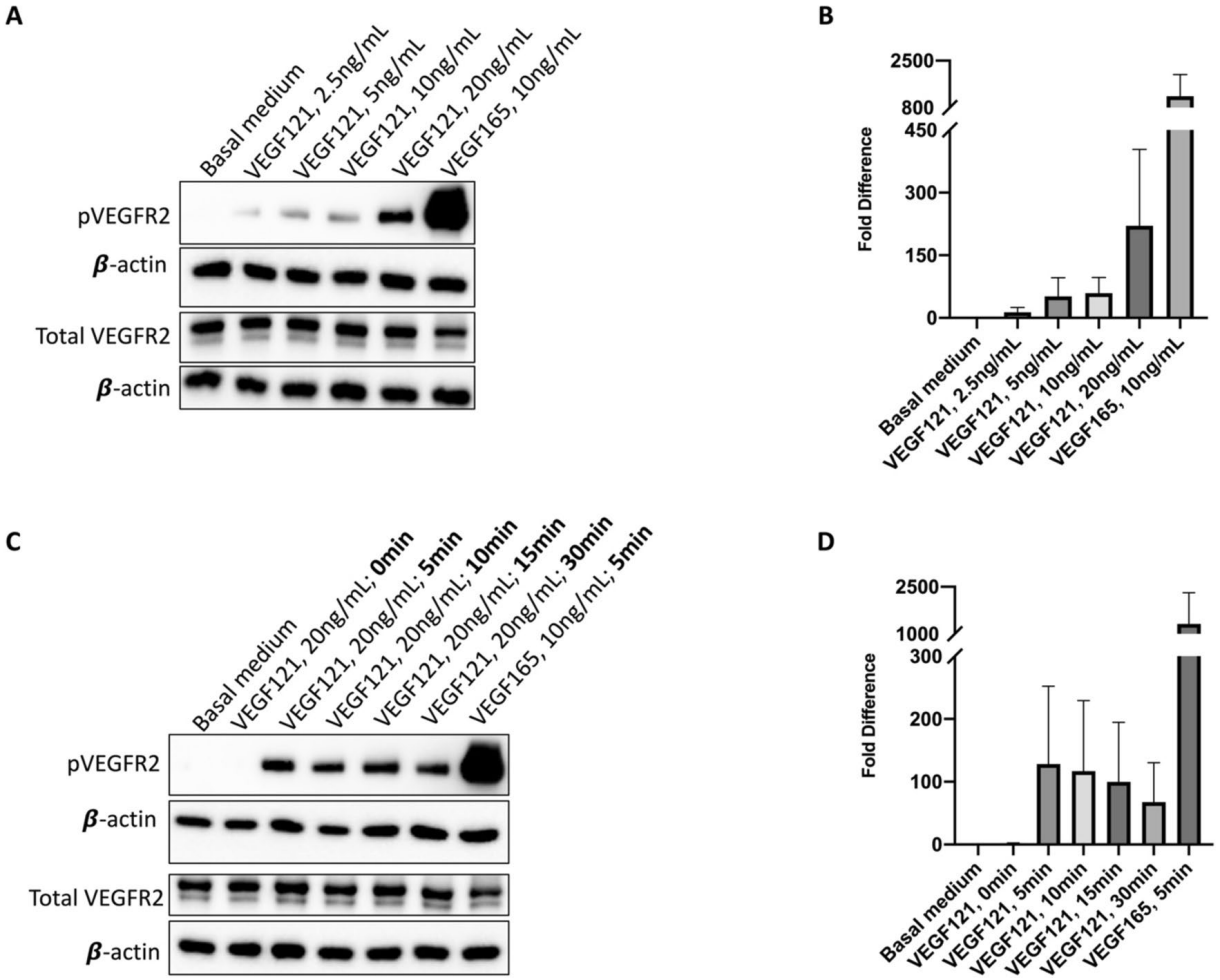
In vitro VEGFR2 activation. In human microvascular lung endothelial cells (HMVEC-L), VEGF121 activated VEGFR2 in a dose dependent manner, although with marked decreased potency compared to VEGF165 in representative immunoblots (**A**, **B**). On an induction study, increased incubation time did not improve VEGF121 receptor binding (**C**, **D**). A representative immunoblot from three independent runs for quality control is pictured (**C**). [Full-length uncropped blots are provided in Supplementary Information File 4. Three-minute exposure, as used in this main figure, is displayed in Supplementary Information File 4A (corresponding to **A**), 4C (corresponding to **C**), while additional 30 s exposures merged with correlating molecular weight markers, are displayed in Supplementary Information File 4B (corresponding to **A**), 4D (corresponding to **B**)]. The β-actin loading control from each membrane is displayed under the image of the primary antibody of interest.

In vitro activation and induction studies were performed using human microvascular lung endothelial cells (HMVEC-L) to determine the role of the HBD in downstream VEGF and potential subsequent paracrine signaling. Increasing doses of VEGF121, the human isoform of VEGF lacking the HBD, induced a dose-dependent activation of VEGFR2, as measured by the ratio of phosphorylated (p-Y1175) to total VEGFR2 after five minutes (Fig. 6A,B, Supplemental Fig. S4A,B). However, VEGF165, which contains the HBD, stimulated VEGFR2 activation to a much higher degree than VEGF121 at a lower dose. The efficacy of both mouse and human isoforms of VEGF to activate VEGFR2 in HMVEC-L was verified, and again demonstrated significantly more robust receptor activation with VEGF164/5 treatment at a lower dose compared to VEGF120/1 (Supplemental Fig. S5).

To assess whether the smaller and more diffusible VEGF121 isoform may require more time to associate with its receptor, an induction study was performed in which HMVEC-L were incubated with VEGF121 for increasing durations of time. Additional time for receptor association did not significantly increase VEGFR2 activation compared to VEGF165 activation of VEGFR2, which was substantially more robust than any time point for VEGF121 (Fig. 6C,D). However, on an extended induction study, VEGF121 did demonstrate strongest receptor activation at 10 min, sustained up to 30 min, with subsequent decreased VEGFR2 activation at time points longer than 30 min (Supplemental Fig. S4C,D). Together, these data suggest that the VEGF HBD is required for potent and timely receptor activation in pulmonary microvascular endothelial cells, which potentially correlate to accelerated CLG with VEGF164 treatment in vivo.

## Discussion

Prior work from our group demonstrated that exogenous, systemic VEGF164 therapy accelerates CLG in a murine model^13,14^. However, heparin, a commonly used anticoagulant in the clinical setting, impairs VEGF-mediated pulmonary growth and development^15^. VEGF164/165 and VEGF188/189 are well-known to interact with exogenous heparins and endogenous heparans via their HBD. Prior clinical studies of systemic heparin infusion have demonstrated consistent downregulation of circulating plasma VEGF^21,22^. As such, in this current study, we sought to circumvent the VEGF-heparin interaction by assessing the efficacy of a non-heparin-binding VEGF isoform on CLG.

Our results indicate that while both VEGF120, which lacks the HBD, and VEGF164, which contains the HBD, increased lung volume and total lung capacity in a dose-dependent manner in mice, only VEGF164 produced functionally relevant improvements. VEGF164 significantly increased treadmill exercise tolerance after pneumonectomy, while VEGF120 did not. Similarly, on the histological level, VEGF164 increased parenchymal volume, alveolar volume, and septal surface area, which represent increased functional area available for gas exchange. VEGF120 produced minimal improvements in exercise tolerance or morphometrics compared to controls. On tissue analysis, VEGF164 consistently induced more pronounced effects on VEGF signaling than VEGF120, with consequent increases in activated VEGFR2, AKT-T308, and MAPK/ERK (p44/42).

In the lungs, VEGF originates from the mesenchymal and alveolar epithelial cells, and paracrine signaling drives endothelial cell proliferation and branching of the pulmonary vasculature^5–7^. However, endothelial cell growth is thought to be the primary driver of pulmonary development through microvascular endothelial cell-mediated “angiocrine” signaling^23^. Downstream endothelial VEGF signaling through AKT, MAPK/ERK, and other various cascades leads to cell survival, proliferation, and paracrine signaling to alveolar epithelial cells^24–26^. Our group has previously shown that provision of exogenous VEGF to HMVEC-L drives human bronchial epithelial cell proliferation in co-culture, and that addition of a neutralizing antibody to Hb-EGF abrogates this effect^27^. Our current study demonstrates that EGFR activation following VEGF treatment in a CLG model is more pronounced than VEGFR2 activation (Fig. 4B,F). IHC demonstrating that VEGF affects total pulmonary cell proliferation more than specific endothelial cell proliferation (Fig. 5B,C) provides further evidence that VEGF-mediated paracrine signaling may be the driver of accelerated pulmonary growth. Similar to effects observed on pulmonary function and TETT, VEGF-mediated signaling and subsequent pulmonary cell proliferative effects were more robust with VEGF164 treatment than VEGF120 treatment. Increased activation of VEGFR2 in cultured HMVEC-L in response to VEGF164/5 relative to VEGF120/1 also suggests that the VEGF HBD is required for potent VEGFR2 signaling (Fig. 6, Supplemental Fig. S4).

Our group has previously demonstrated that the process of CLG after left pneumonectomy approaches completion by POD eight to ten^13^. Exogenous VEGF therapy consistently accelerates CLG to completion by POD4^13,14,27^. However, while we have already characterized that exogenous VEGF modulates VEGFR2 activation and paracrine signaling through EGFR, P-Y1175-VEGFR2-mediated downstream signaling at POD4 in VEGF-treated mice has not previously been investigated. While a limitation of this study is that there is a significant degree of variability between biological replicates, it is important to note that, on average, VEGF164 treatment produced more significant effects in signaling downstream of VEGFR2, as well as in activating EGFR (Fig. 4B,F). Significant downregulation of pAKT-S473 and NRP2 is unique to VEGF164-treated lung tissue and is not present in control or VEGF120-treated lung tissue (Fig. 4B,D), which presumably have not completed CLG by POD4. As such, modulation of pAKT-S473 and NRP2 may represent early molecular signals of pulmonary growth completion.

The role of neuropilins (NRPs) in lung development has not been thoroughly evaluated. However, neuropilin-1 (NRP1) is known to be essential for VEGF-stimulated heterodimerization of NRP1 with VEGFR2 and downstream endothelial cell migration signaling^28^. Previously, VEGF120 was believed to be unable to bind NRP due to the lack of the HBD (exons 6 and 7), which also contains the NRP-binding site. New evidence, including the resolved crystal structures of NRP and its bound state with VEGF, revealed that NRPs interact with VEGF exons 5, 7, and 8, the former and latter of which are still present on VEGF120/1^29,30^. However, the binding affinity of VEGF120 to NRP is much less than that of VEGF164^29^, and is inconsistent across studies^30^. The pattern of decreased affinity for VEGF120 relative to VEGF164 is similar to the decreased activation of VEGFR2 observed in our in vitro assays with VEGF121 (Fig. 6), despite exogenous VEGF treatment having little effect on pulmonary expression of NRP1 (Fig. 4C,D). In addition, interactions with cell surface and extracellular matrix (ECM) heparans further complicate the system. Interestingly, in cell-free kinetics studies, NRP1 enhanced VEGF165 interaction with VEGFR2, but not when endogenous heparan sulfate proteoglycans (HSPGs) were missing^31^. This suggests that NRPs and endogenous HSPGs may complex with VEGF and VEGR2 to enhance receptor-ligand affinity and promote downstream signaling. In this study, we demonstrated that VEGF120/121 has reduced potency for activating VEGFR2 in vitro and in vivo compared to VEGF164/165. We therefore postulate that effective interactions with NRPs, and perhaps with endogenous HSPGs on the cell surface or ECM, may be enhanced by the presence of the HBD. Therefore, the VEGF HBD may be essential to effective exogenous VEGF/VEGFR2 binding on endothelial cells, downstream signaling, and subsequent CLG. Future research endeavors may further focus on the crosstalk between the VEGF HBD, HRPs and HSPGs by elimination or inactivation of NRPs, HSPGs, or both sequentially in both in vitro and in vivo systems.

This study should be considered in light of its limitations, chief among which are the lack of equivalency between a CLG model and CDH-associated PH, lack of additional post-operative days evaluating VEGF120 treatment, and lack of statistical significance in much of the lung tissue protein analysis. In addition, commercially available recombinant murine VEGF may have batch-to-batch variation, which may have affected the efficacy of the proteins used therapeutically. While CLG and pulmonary development are driven by similar mechanical stress responses and molecular patterning, the two processes are not identical^11,12^. CDH is further driven by other genetic and diaphragmatic developmental deficiencies that may crosstalk and interfere with normal pulmonary development^32^. VEGF120 treatment failed to produce significant effects on exercise tolerance or molecular signaling outcomes in our model, but significantly increased TLC, and trended towards increased lung volume, activated ERK, activated EGFR, and pulmonary total cell and endothelial cell proliferation compared to controls. These data suggest that VEGF120 may accelerate CLG, but to a lesser degree than VEGF164. Expeditious pulmonary growth is essential for infant survival following surgical repair of CDH, as prolonged dependence on invasive supportive care such as ECMO or even mechanical ventilation are associated with worse outcomes and lower survival^2^. Therefore, any therapeutic that accelerates lung growth may be worthwhile to pursue. Nevertheless, it is difficult to draw further conclusions on the efficacy of VEGF120 to accelerate lung growth without further data, and difficult to predict if such marginal effects may have a clinical impact. From a technical perspective, this manuscript is limited by VEGF120/1 and VEGF164/5 being commercially available proteins made in different organisms and purified using different methods. Although the resulting total impurities of the preparation methods were comparable (< 3% versus < 4% for VEGF164 and VEGF120, respectively), the observed differences in this manuscript may at least partially be accounted for by the differences in preparation, impurities, and potential immune or other extramural effects caused by exogenous protein administration.

In summary, this study provides additional evidence for the therapeutic potential of VEGF164/165. In addition to consistently demonstrating increased alveolarization on lung morphometric analysis, we now provide data to indicate improved functional outcomes with VEGF164 treatment after left pneumonectomy, as evidenced by improved pulmonary function and exercise tolerance. Further, the importance of the heparin-binding domain in potentiating downstream VEGF-mediated signaling and pulmonary proliferative effects is established in this study. However, the essentiality of the VEGF HBD raises concern for the translatability of VEGF164/165 as a therapy for children with pulmonary hypoplasia who require anticoagulation with heparin. While VEGF120/121 may avoid deleterious interactions with heparin that impair pulmonary proliferation, it lacks the potency to drive clinically relevant accelerated lung growth and development. Alternative therapeutics for pulmonary hypoplasia that either do not interact with heparin, or alternative anticoagulants which do not interact with angiogenic factors such as VEGF, deserve further expedient investigation to treat this often-fatal disease.

## Methods

### Surgical animal model and experimental groups

All procedures were carried out according to the National Institutes of Health Guide for the Care and Use of Laboratory Animals and approved by the Institutional Animal Care and Use Committee at Boston Children’s Hospital and in compliance with ARRIVE guidelines. Eight-week-old, male, C57BL/6J mice were anesthetized with ketamine (80–100 mg/kg) and xylazine (10–12.5 mg/kg) via intraperitoneal injection, orotracheally intubated, then placed on a mouse ventilator (HSE-HA Minivent, Harvard Apparatus, Holliston, MA) at 150 breaths/min. Mice then underwent left pneumonectomy as previously described^33^. Post-operatively, three milliliters of normal saline was injected subcutaneous for fluid resuscitation. Analgesia was maintained with subcutaneous, sustained-release buprenorphine immediately after the procedure and every 48 h thereafter. After pneumonectomy, mice were randomized to three groups of daily intraperitoneal injections for four days starting on POD 0: VEGF164 (0.25 mg/kg, 0.5 mg/kg) (Z02690-1, GenScript, Piscataway, NJ), equimolar VEGF120 (0.182 mg/kg, 0.363 mg/kg) (Z02779-1, GenScript, Piscataway, NJ), or isovolumetric normal saline as a vehicle control. Of note, these recombinant proteins were devoid of any carrier proteins. Doses were determined based on prior studies from our laboratory^13,14,27^, and within the range of VEGF or similar exogenous angiogenic protein administration in the published literature^34–40^. An additional cohort of mice (N = 2–3 per group) either received an inactive form of VEGF164 or VEGF120, created by boiling the VEGF at 95 °C for 30 min. Methods for heat inactivation of VEGFs and other angiogenic factors have previously been validated^41–43^. Mice treated with inactive VEGFs underwent assessment of hematocrit and other organs, described below, as well as lung volume measurement and pulmonary function testing. Of the active treatment groups, one cohort underwent lung volume measurement, pulmonary function testing, and histological analysis (N = 68), while another cohort underwent treadmill exercise tolerance testing and lung tissue protein analysis (N = 35), described below. All analyses were performed on POD4, which is the day by which VEGF-accelerated compensatory lung growth was previously deemed to be complete^13,14^.

### Hematocrit analysis

Hematocrit levels were analyzed in a small cohort of mice (N = 2–3 per group). On POD4, blood was collected via venipuncture from the inferior vena cava into a heparinized microcapillary tube. Tubes were centrifuged at 2000×*g* for 20 min at 4 °C. The hematocrit was reported as a percentage determined by measuring the ratio of red blood cells to total column height after centrifugation.

### Pulmonary function testing

Mice were anesthetized with ketamine (80–100 mg/kg) and xylazine (10–12.5 mg/kg) and paralyzed with pancuronium (0.8 mg/kg) via intraperitoneal injection for tracheostomy and pulmonary function testing (PFT). Briefly, the trachea was exposed via a midline neck incision, an anterior tracheotomy was made, and the trachea was intubated with a beveled 20-gauge hollow needle to connect to a Flexivent system (SCIREQ, Montreal, Canada). Compliance and total lung capacity (TLC) were measured as previously described using the Flexivent system, in which the machine is programmed to ventilate the subject with 100% oxygen in a closed system, followed by complete degassing and euthanasia via tracheal occlusion^44^. Additional details of the Flexivent system pulmonary function maneuvers are provided in the Supplemental Methods. TLC and compliance were normalized to body weight. Lung tissue was then permanently fixed for morphometric analysis and immunohistochemistry (IHC).

### Organ harvest and volume measurement

Following PFT, the remaining right lung was removed and instilled with 10% formalin at 35 cmH_2_O. Lung volume was measured by water displacement and normalized to body weight^45^. Other organs were removed, weighed, and normalized to body weight. Following lung inflation, the trachea was ligated with a 4–0 silk suture, and the lung was submerged in 10% formalin at 4 °C for 24 h before transfer to 70% ethanol. All specimens were then processed and paraffin embedded for histologic analysis.

### Morphometric analysis

Hematoxylin and eosin (H&E)-stained lung sections were assessed by qualitative microscopy based on principles of stereology (N = 5 per treatment group). In brief, 17 lung fields per section at 200X magnification were selected based on systematic uniform random sampling for morphometric analysis using a previously validated point and intersection counting method^46,47^. This technique yielded values for parenchymal and alveolar volume, septal surface area, and mean septal thickness, of which all volume and area measurements were normalized to body weight.

### Treadmill exercise tolerance testing

Two days prior to surgery, mice were placed on a rodent treadmill with attached shock grid (IITC Life Science, Woodland Hills, CA) for baseline treadmill exercise tolerance testing (TETT). They first underwent an acclimation trial on the stationary treadmill with the shock grid on for five minutes, followed by five minutes on the treadmill at 5 m/min. The TETT consisted of 10 min of acceleration starting at 5 m/min up to 14 m/min, followed by a sustained period of 15 m/min running until exhaustion. Exhaustion was defined as remaining on the shock grid for more than five seconds despite receiving multiple low-voltage shocks. Exercise time and distance were recorded. Repeat TETT was performed on POD4. Absolute change in exercise tolerance was obtained by subtracting the baseline time or distance from the POD4 time or distance.

### Pulmonary protein analysis

Following TETT, mice were euthanized via CO2 asphyxiation. Right lung tissue was then collected and flash frozen in liquid nitrogen for protein analysis. Approximately 30-g lung specimens were homogenized in radioimmunoprecipitation assay (RIPA) buffer containing protease and phosphatase inhibitors (Thermo Fischer Scientific, Waltham, MA) then centrifuged. Supernatant containing protein lysates were resuspended in 1X Laemmli buffer, resolved on SDS 4–10% PAGE, and transferred onto polyvinyl difluoride (PVDF) membranes (Merck Millipore, Darmstadt, Germany). Membranes were blocked in 5% nonfat milk in tris-buffered saline with Tween 20 (TBST) for one hour and incubated with 1:1000 dilution primary antibodies (anti-VEGF_120/164_, -Hb-EGF [R&D Systems, Minneapolis, MN]; -p-Y1175-VEGFR2, -VEGFR2, -NRP1, -NRP2, -p-p44/42 (pERK), -p44/42 (ERK), -p-AKT-S473, -p-AKT-T308, -AKT, -p-1068-EGFR, -EGFR [Cell Signaling Technology, Davers, MA]) in 5% nonfat milk-TBST at 4 °C overnight. Blots were probed with β-actin antibody (Sigma-Aldrich, St. Louis, MO) as a loading control. Membranes were washed three times with TBST, incubated with the appropriate horseradish peroxidase-conjugated secondary antibody (anti-rabbit or anti-goat IgG [R&D Systems, Minneapolis, MN] at 1:2000 dilution in 5% nonfat milk-TBST for one hour at room temperature, and developed by enhanced chemiluminescence reagents (Bio-Rad, Hercules, CA) on a ChemiDocTM Touch System imager (Bio-Rad, Hercules, CA). Quantification of signals was performed with Image Lab software v.6.0.1 (Bio-Rad, Hercules, CA).

### Immunohistochemistry

Total pulmonary cell proliferation and pulmonary endothelial cell proliferation on formalin-fixed paraffin-embedded lung sections was assessed by immunofluorescence as previously described^14,27,44^. Sections were deparaffinized with xylene and progressively rehydrated in decreasing concentrations of ethanol, ending in phosphate-buffered saline (PBS). Epitope retrieval was performed with a citrate-based unmasking solution (Vector Laboratories, Burlingame, CA) at 120 °C in a pressurized chamber (Decloaking Chamber, Biocare Medical, Pacheco, CA). Following epitope retrieval, sections were permeabilized three times with 0.05% Triton-X in phosphate-buffered saline (PBST) for 10 min, followed by incubation in blocking solution (1% bovine serum albumin in PBST) for 30 min. Primary antibodies, rat anti-Ki67 (Invitrogen, Carlsbad, CA), and rabbit anti-ERG (Abcam, Cambridge, MA) were prepared in blocking solution and incubated overnight at 4 °C. Following primary antibody incubation, slides were again washed three times with PBST for 10 min, followed by incubation with secondary antibodies, Alexa-Fluor-conjugated donkey anti-rat (Abcam, Cambridge, MA) and donkey anti-rabbit (Invitrogen, Carlsbad, CA) IgG, for two hours at room temperature. Sections were counterstained for nuclear DAPI for five minutes, then washed again three times with PBST for 10 min. Slides were dried and mounted with Fluoromount (Thermo Fischer Scientific, Waltham, MA).

Sections of control, VEGF120-, and VEGF164-treated lungs were assessed with confocal microscopy (LSM880Fast, Zeiss, Jena, Germany) at 200X magnification as previously described^14,27,44^. For each specimen, cell counting was performed using 10 random, 7-tiled high-power fields (HPF) spanning the entire right lung. Cells were quantified with Zeiss ZEN Blue Image Analysis Software (Jena, Germany). Proliferating endothelial cells were determined as those co-stained with DAPI, Ki67, and ERG, while total proliferating cells were determined as those co-stained with at least DAPI and Ki67. Percent endothelial cell proliferation was calculated by normalizing the number of proliferating endothelial cells (DAPI/Ki67/ERG) against total endothelial cells (DAPI/ERG), while percent cell proliferation was calculated by normalizing the number of proliferating cells (DAPI/Ki67) against total cells (DAPI).

An additional small cohort (N = 2 per group) of control, VEGF120- and VEGF-164 treated non-proliferative organs were collected and formalin-fixed paraffin-embedded for sectioning. These organs included the liver, right kidney, and gastrocnemius skeletal muscle. Organ sections underwent immunofluorescence staining as described above, also with co-staining of Ki-67 and ERG with DAPI nuclear counterstain. Following staining, organs were assessed with confocal microscopy at 200 × magnification as described above for each organ, and representative images were captured using 7-tiled HPF spanning the entire section.

### Lung endothelial cell culture

Human microvascular lung endothelial cells (HMVEC-L) (Lonza, Morristown, NJ) were plated at 70% confluence on gelatin-coated plates in endothelial growth media (EGM-2 + 5% fetal bovine serum [Lonza, Morristown, NJ]). After ensuring cell attachment, cells were starved overnight in basal medium (EBM-2 medium + 0.5% fetal bovine serum [Lonza, Morristown, NJ]) at 37 °C. After 12 h, cells were washed and treated for the activation or induction assays as described below.

#### VEGFR2 activation assay

After overnight starvation, cells were treated with basal medium, basal medium + increasing doses of VEGF121 (2.5, 5, 10, 20, 100, 200 ng/mL), or VEGF165 (10 ng/mL) as a positive control for five minutes at 37 °C. The media was removed, and cells were lysed on ice with RIPA buffer containing protease and phosphatase inhibitors (Thermo Fischer Scientific, Waltham, MA) then centrifuged. The supernatant was then resuspended in 1X Laemmli buffer (Boston Bio Products, Ashland, MA) and analyzed for phosphorylated and total VEGFR2 (P-Y1175-VEGFR2, tVEGFR2) with Western immunoblot.

#### VEGF120 induction assay

After overnight starvation, cells were treated with basal medium, basal medium + VEGF121 (20 ng/mL), or VEGF165 (10 ng/mL). Treatment was allowed to incubate at 37°C for 0, 5, 10, 15, 30 minutes, and 1, 2, 4 h for VEGF121, or the standard 5 mins for basal medium (negative control) and VEGF165 (positive control). The media was then removed, and cells were lysed on ice with RIPA buffer containing protease and phosphatase inhibitors (Thermo Fischer Scientific, Waltham, MA) then centrifuged. The supernatant was then resuspended in 1X Laemmli buffer (Boston Bio Products, Ashland, MA) and analyzed for phosphorylated and total VEGFR2 (P-Y1175-VEGFR2, tVEGFR2) with Western immunoblot.

### Statistical analysis

All outcomes except TETT were compared across groups using a one-way analysis of variance (ANOVA) with results expressed as mean ± standard error (SE). For TETT, groups were compared by nonparametric analysis of covariance (ANCOVA)^48^, with adjustment for pre-operative outcome. Graphs of raw data and sensitivity analysis are provided in Supplemental Fig. S6, and further discussion of the rationale and methodology of nonparametric ANCOVA analysis for TETT is provided in the Supplemental Methods. Results for TETT are reported as median and interquartile range (IQR), with *P* value from nonparametric ANCOVA. For all analysis, a *P* value of < 0.05 was considered statistically significant and adjustment for multiple pairwise comparisons (control vs. VEGF120; control vs. VEGF164; VEGF120 vs. VEGF164) was made by Holm-Sidak correction. All analyses were performed with GraphPad Prism v.8 (La Jolla, CA) and SAS v.9.4 (Cary, NC).

## Supplementary Information

Supplementary Information Files.

Supplementary Figures.

Supplementary Method.

## Abbreviations

CDH: Congenital diaphragmatic hernia
CLG: Compensatory lung growth
ECMO: Extracorporeal membrane oxygenation
HBD: Heparin-binding domain
Hb-EGF: Heparin-binding epithelial growth factor
HMVEC-L: Human microvascular lung endothelial cells
HPF: High power field
H&E: Hematoxylin and eosin
IHC: Immunohistochemistry
PBS: Phosphate-buffered saline
PBST: Phosphate-buffered saline with 0.05% Triton-X
PFT: Pulmonary function test(ing)
POD: Post-operative day
PVDF: Polyvinyl difluoride
PH: Pulmonary hypoplasia
pEGFR: Phosphorylated epithelial growth factor receptor
pHTN: Pulmonary hypertension
pVEGFR2: Phosphorylated vascular endothelial growth factor receptor 2
RIPA: Radioimmunoprecipitation assay
SDS-PAGE: Sodium dodecyl sulfate–polyacrylamide gel electrophoresis
TETT: Treadmill exercise tolerance testing
TBST: Tris-buffered saline with Tween-20
TLC: Total lung capacity
VEGF: Vascular endothelial growth factor
tEGFR: Total epithelial growth factor receptor
tVEGFR2: Total vascular endothelial growth factor receptor 2

## References

[1] Tsao K, Allison ND, Harting MT, Lally PA, Lally KP (2010). Congenital diaphragmatic hernia in the preterm infant. Surgery..

[2] Seetharamaiah R, Younger JG, Bartlett RH, Hirschl RB (2009). Factors associated with survival in infants with congenital diaphragmatic hernia requiring extracorporeal membrane oxygenation: A report from the Congenital Diaphragmatic Hernia Study Group. J. Pediatr. Surg..

[3] Chang R, Andreoli S, Ng YS, Truong T, Smith SR, Wilson J (2004). VEGF expression is downregulated in nitrofen-induced congenital diaphragmatic hernia. J. Pediatr. Surg..

[4] van der Horst IW, Rajatapiti P, van der Voorn P, van Nederveen FH, Tibboel D, Rottier R (2011). Expression of hypoxia-inducible factors, regulators, and target genes in congenital diaphragmatic hernia patients. Pediatr. Dev. Pathol..

[5] Acarregui MJ, Penisten ST, Goss KL, Ramirez K, Snyder JM (1999). Vascular endothelial growth factor gene expression in human fetal lung in vitro. Am. J. Respir. Cell Mol. Biol..

[6] Aman J, Bogaard HJ, Vonk NA (2016). Why vessels do matter in pulmonary disease. Thorax.

[7] Healy AM, Morgenthau L, Zhu X, Farber HW, Cardoso WV (2000). VEGF is deposited in the subepithelial matrix at the leading edge of branching airways and stimulates neovascularization in the murine embryonic lung. Dev. Dyn..

[8] Lassus P, Ristimaki A, Ylikorkala O, Viinikka L, Andersson S (1999). Vascular endothelial growth factor in human preterm lung. Am. J. Respir. Crit. Care Med..

[9] Thebaud B, Ladha F, Michelakis ED, Sawicka M, Thurston G, Eaton F (2005). Vascular endothelial growth factor gene therapy increases survival, promotes lung angiogenesis, and prevents alveolar damage in hyperoxia-induced lung injury: evidence that angiogenesis participates in alveolarization. Circulation.

[10] Compernolle V, Brusselmans K, Acker T, Hoet P, Tjwa M, Beck H (2002). Loss of HIF-2alpha and inhibition of VEGF impair fetal lung maturation, whereas treatment with VEGF prevents fatal respiratory distress in premature mice. Nat. Med..

[11] Hsia CC (2004). Signals and mechanisms of compensatory lung growth. J. Appl. Physiol..

[12] Voswinckel R, Motejl V, Fehrenbach A, Wegmann M, Mehling T, Fehrenbach H (2004). Characterisation of post-pneumonectomy lung growth in adult mice. Eur. Respir. J..

[13] Sakurai MK, Lee S, Arsenault DA, Nose V, Wilson JM, Heymach JV (2007). Vascular endothelial growth factor accelerates compensatory lung growth after unilateral pneumonectomy. Am. J. Physiol. Lung Cell. Mol. Physiol..

[14] Dao DT, Nandivada P, Vuong JT, Anez-Bustillos L, Pan A, Kishikawa H (2018). Vascular endothelial growth factor accelerates compensatory lung growth by increasing the alveolar units. Pediatr. Res..

[15] Dao DT, Anez-Bustillos L, Ourieff J, Pan A, Mitchell PD, Kishikawa H (2018). Heparin impairs angiogenic signaling and compensatory lung growth after left pneumonectomy. Angiogenesis.

[16] Robinson CJ, Stringer SE (2001). The splice variants of vascular endothelial growth factor (VEGF) and their receptors. J. Cell. Sci..

[17] Galambos C, Ng YS, Ali A, Noguchi A, Lovejoy S, D'Amore PA (2002). Defective pulmonary development in the absence of heparin-binding vascular endothelial growth factor isoforms. Am. J. Respir. Cell. Mol. Biol..

[18] Springer ML, Banfi A, Ye J, von Degenfeld G, Kraft PE, Saini SA (2007). Localization of vascular response to VEGF is not dependent on heparin binding. FASEB J..

[19] Lampropoulou A, Ruhrberg C (2014). Neuropilin regulation of angiogenesis. Biochem. Soc. Trans..

[20] Mohamed AA, Tan SH, Mikhalkevich N, Ponniah S, Vasioukhin V, Bieberich CJ (2010). Ets family protein, erg expression in developing and adult mouse tissues by a highly specific monoclonal antibody. J. Cancer..

[21] East MA, Landis DI, Thompson MA, Annex BH (2003). Effect of single dose of intravenous heparin on plasma levels of angiogenic growth factors. Am. J. Cardiol..

[22] Kapur NK, Shenoy C, Yunis AA, Mohammad NN, Wilson S, Paruchuri V (2012). Distinct effects of unfractionated heparin versus bivalirudin on circulating angiogenic peptides. PLoS ONE.

[23] Woik N, Kroll J (2015). Regulation of lung development and regeneration by the vascular system. Cell. Mol. Life Sci..

[24] Takahashi T, Yamaguchi S, Chida K, Shibuya M (2001). A single autophosphorylation site on KDR/Flk-1 is essential for VEGF-A-dependent activation of PLC-gamma and DNA synthesis in vascular endothelial cells. EMBO J..

[25] Franke TF, Kaplan DR, Cantley LC (1997). PI3K: Downstream AKTion blocks apoptosis. Cell.

[26] Ding BS, Nolan DJ, Guo P, Babazadeh AO, Cao Z, Rosenwaks Z (2011). Endothelial-derived angiocrine signals induce and sustain regenerative lung alveolarization. Cell.

[27] Dao DT, Vuong JT, Anez-Bustillos L, Pan A, Mitchell PD, Fell GL (2018). Intranasal delivery of VEGF enhances compensatory lung growth in mice. PLoS ONE.

[28] Herzog B, Pellet-Many C, Britton G, Hartzoulakis B, Zachary IC (2011). VEGF binding to NRP1 is essential for VEGF stimulation of endothelial cell migration, complex formation between NRP1 and VEGFR2, and signaling via FAK Tyr407 phosphorylation. Mol. Biol. Cell..

[29] Parker MW, Xu P, Li X, Vander Kooi CW (2012). Structural basis for selective vascular endothelial growth factor-A (VEGF-A) binding to neuropilin-1. J. Biol. Chem..

[30] Sarabipour S, Mac GF (2018). VEGF-A121a binding to Neuropilins: A concept revisited. Cell. Adher. Migr..

[31] Teran M, Nugent MA (2015). Synergistic binding of vascular endothelial growth factor-A and its receptors to heparin selectively modulates complex affinity. J. Biol. Chem..

[32] Wynn J, Yu L, Chung WK (2014). Genetic causes of congenital diaphragmatic hernia. Semin. Fetal Neonatal. Med..

[33] Sakurai MK, Greene AK, Wilson J, Fauza D, Puder M (2005). Pneumonectomy in the mouse: Technique and perioperative management. J. Invest. Surg..

[34] O'Reilly MS, Boehm T, Shing Y, Fukai N, Vasios G, Lane WS (1997). Endostatin: An endogenous inhibitor of angiogenesis and tumor growth. Cell.

[35] Rennel ES, Hamdollah-Zadeh MA, Wheatley ER, Magnussen A, Schuler Y, Kelly SP (2008). Recombinant human VEGF165b protein is an effective anti-cancer agent in mice. Eur. J. Cancer..

[36] Hua J, Spee C, Kase S, Rennel ES, Magnussen AL, Qiu Y (2010). Recombinant human VEGF165b inhibits experimental choroidal neovascularization. Invest. Ophthalmol. Vis. Sci..

[37] Waldner MJ, Wirtz S, Jefremow A, Warntjen M, Neufert C, Atreya R (2010). VEGF receptor signaling links inflammation and tumorigenesis in colitis-associated cancer. J. Exp. Med..

[38] Ludin A, Sela JJ, Schroeder A, Samuni Y, Nitzan DW, Amir G (2013). Injection of vascular endothelial growth factor into knee joints induces osteoarthritis in mice. Osteoarthr. Cartil..

[39] Verheyen A, Peeraer E, Lambrechts D, Poesen K, Carmeliet P, Shibuya M (2013). Therapeutic potential of VEGF and VEGF-derived peptide in peripheral neuropathies. Neuroscience.

[40] Liu Y, Shen J, Fortmann SD, Wang J, Vestweber D, Campochiaro PA (2017). Reversible retinal vessel closure from VEGF-induced leukocyte plugging. JCI Insight..

[41] Roberts WG, Palade GE (1995). Increased microvascular permeability and endothelial fenestration induced by vascular endothelial growth factor. J. Cell. Sci..

[42] McCloskey DP, Hintz TM, Scharfman HE (2008). Modulation of vascular endothelial growth factor (VEGF) expression in motor neurons and its electrophysiological effects. Brain Res. Bull..

[43] Zhang SX, Wang JJ, Gao G, Parke K, Ma JX (2006). Pigment epithelium-derived factor downregulates vascular endothelial growth factor (VEGF) expression and inhibits VEGF-VEGF receptor 2 binding in diabetic retinopathy. J. Mol. Endocrinol..

[44] Ko VH, Yu LJ, Dao DT, Li X, Secor JD, Anez-Bustillos L (2020). Roxadustat (FG-4592) accelerates pulmonary growth, development, and function in a compensatory lung growth model. Angiogenesis.

[45] Scherle W (1970). A simple method for volumetry of organs in quantitative stereology. Mikroskopie.

[46] Ochs M, Muhlfeld C (2013). Quantitative microscopy of the lung: A problem-based approach. Part 1: Basic principles of lung stereology. Am. J. Physiol. Lung. Cell. Mol. Physiol..

[47] Muhlfeld C, Ochs M (2013). Quantitative microscopy of the lung: A problem-based approach. Part 2: Stereological parameters and study designs in various diseases of the respiratory tract. Am. J. Physiol. Lung. Cell. Mol. Physiol..

[48] Quade D (1967). Rank analysis of covariance. JASA..

