# Supplementary material for: Investigation of the mechanisms of VEGF-mediated compensatory lung growth: the role of the VEGF heparin-binding domain
Source: Sci Rep. 2021 Jun 4;11:11827. doi: 10.1038/s41598-021-91127-0 (PMC8178332; doi:10.1038/s41598-021-91127-0)

Investigation of the mechanisms of VEGF-mediated compensatory lung growth: The role of the VEGF heparin-binding domain

Lumeng J. Yu[1,2], Victoria H. Ko[1,2], Duy T. Dao[1,2], Jordan D. Secor[1,2], Amy Pan[1,2], Bennet S. Cho[1,2], Paul D. Mitchell[3], Hiroko Kishikawa[1,2], Diane R. Bielenberg[1], Mark Puder[1,2]*

[1] Vascular Biology Program, Boston Children's Hospital, Harvard Medical School, Boston, MA 02115, USA
[2] Department of Surgery, Boston Children's Hospital, Harvard Medical School, 300 Longwood Ave, Fegan 3, Boston, MA 02115, USA
[3] Institutional Centers for Clinical and Translational Research, Boston Children's Hospital, Boston, MA 02115, USA
*Correspondence to: mark.puder@childrens.harvard.edu

Supplementary Information File 1

(Corresponds to Figure 4A)

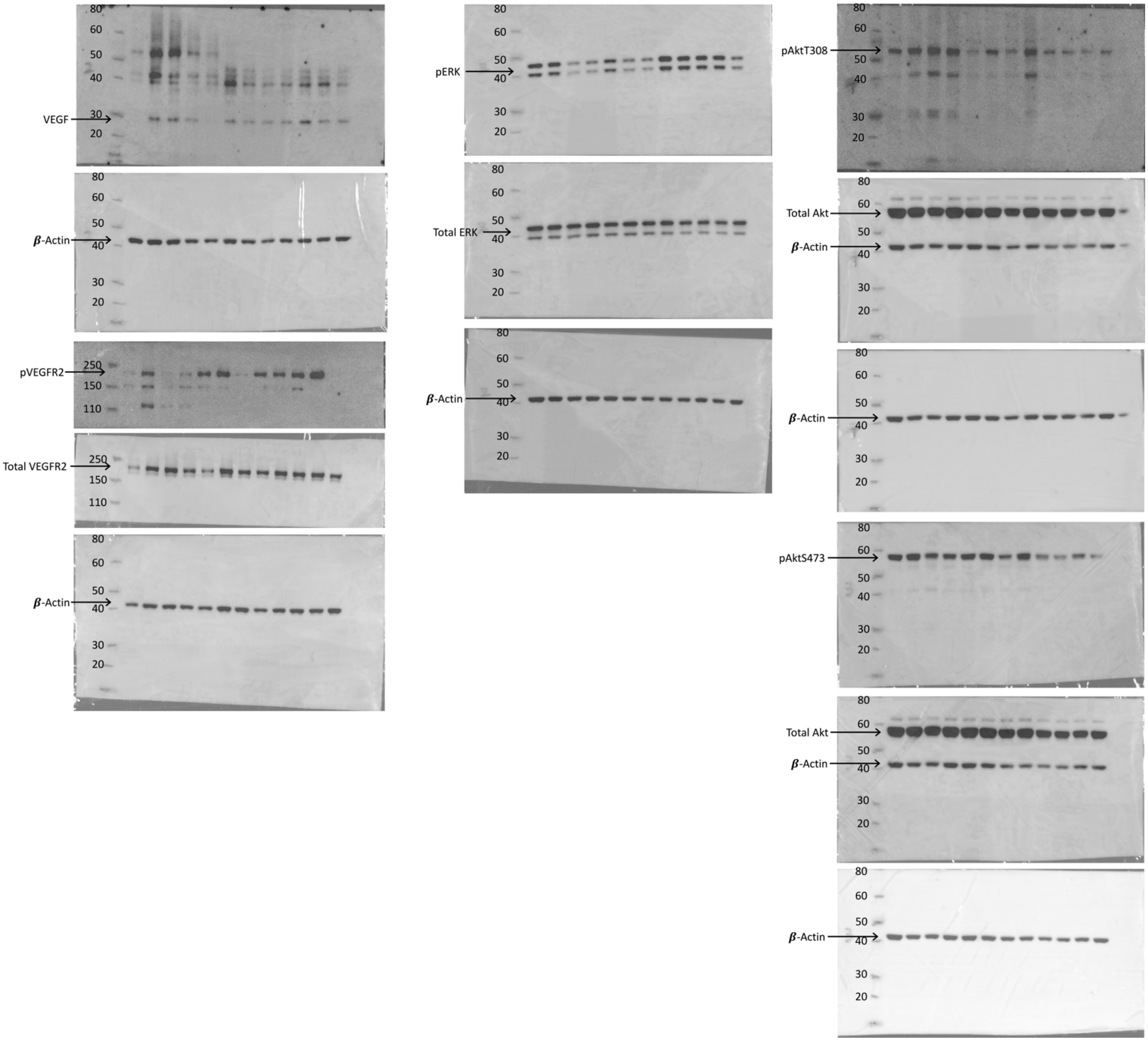

Supplementary Information File 2

(Corresponds to Figure 4C)

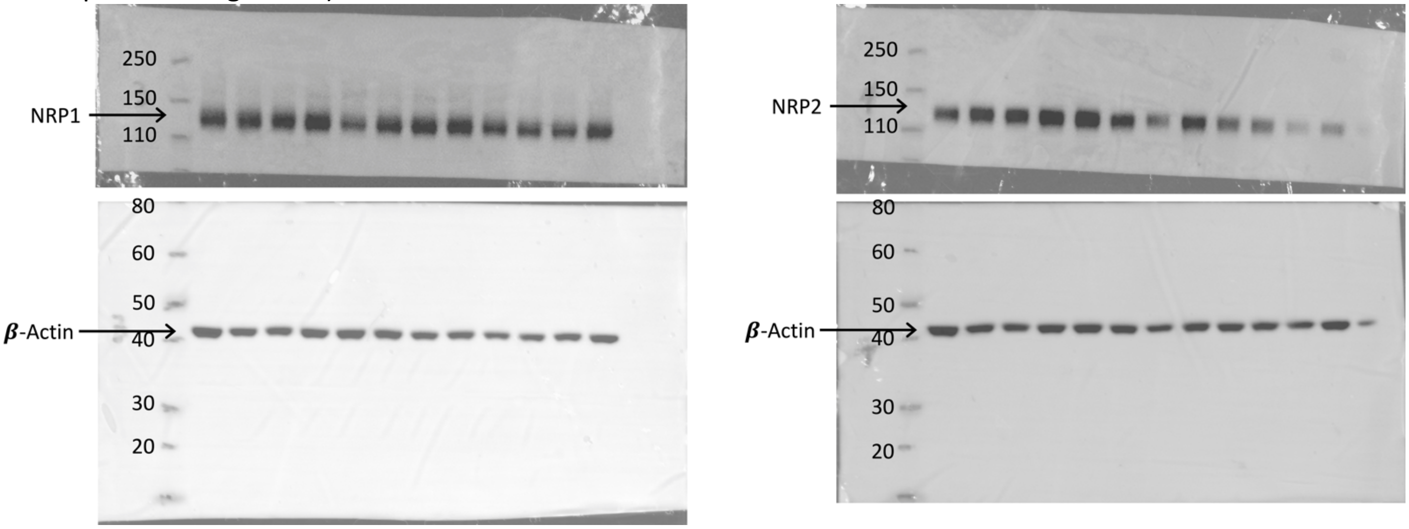

Supplementary Information File 3
(Corresponds to Figure 4E)

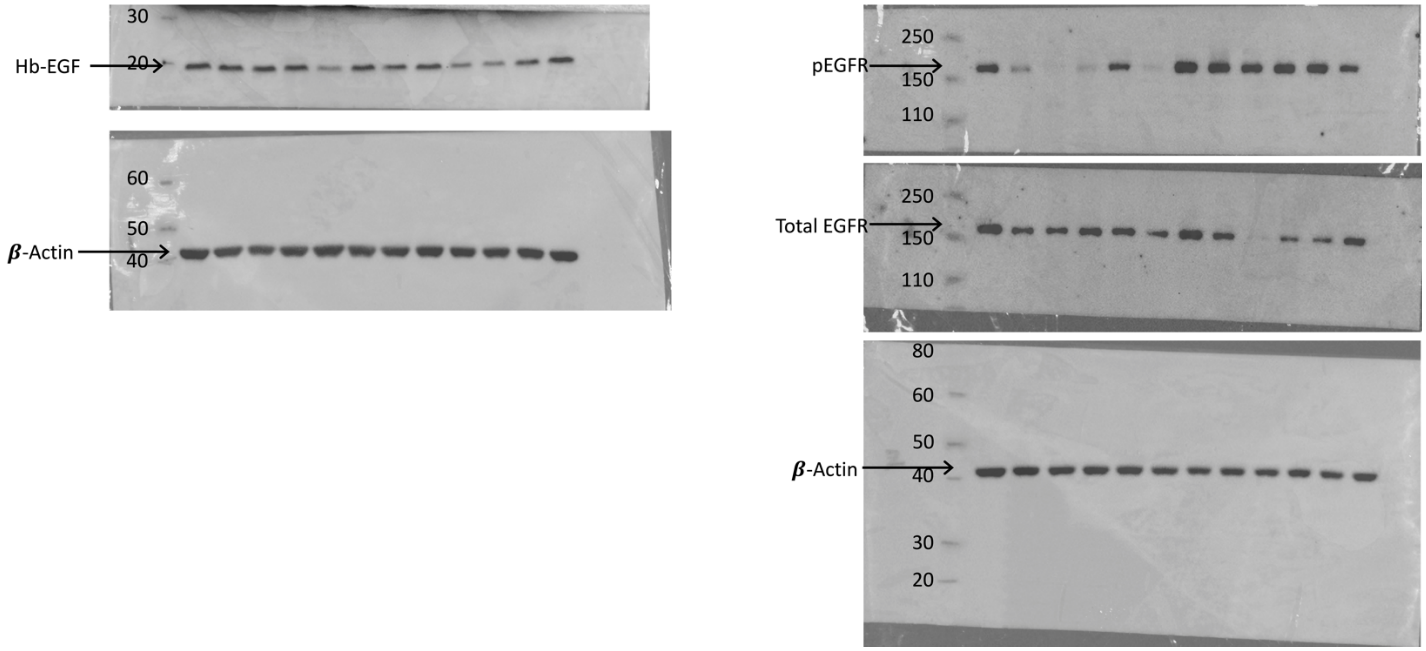

Supplementary Information File 4

(A, B Corresponds to Figure 6A)(C, D Corresponds to Figure 6C)

A, C – 3 minute exposure

B, D – 30 second Exposure

E – Blot D was stripped and reprobed with total VEGFR2 primary antibody to demonstrate pattern near-identical to total VEGFR2 blot performed simultaneously using the same lysate on a different gel. As identical technique was performed by the same operator for all blots in Figure 6/Supplementary Information File 4, no addition striping/reprobing experiments were performed to verify total VEGFR2 results.

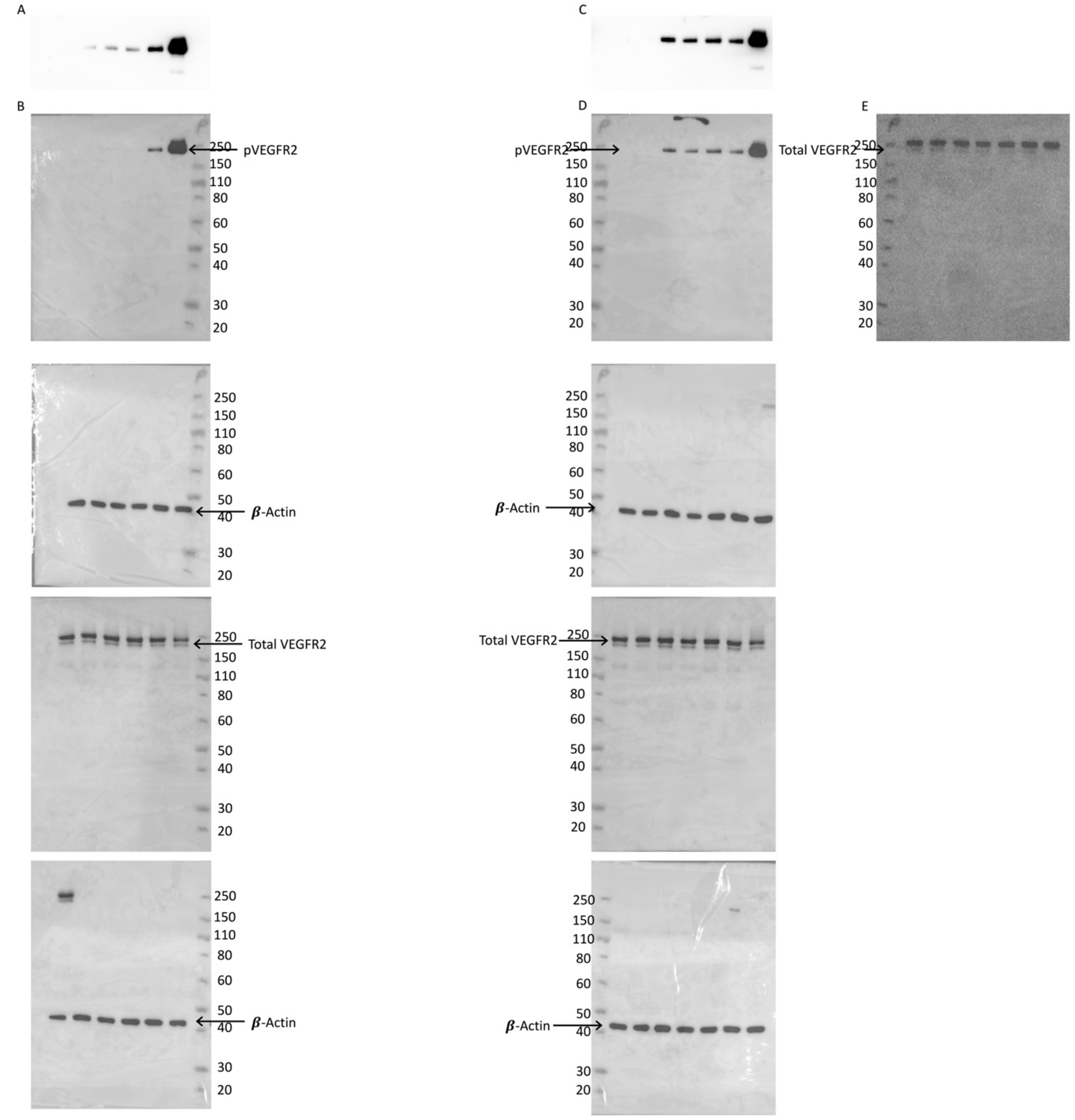

Supplementary Information File 5

(Corresponds to Supplemental Figure 4A (left side of marker), C (right side of marker))

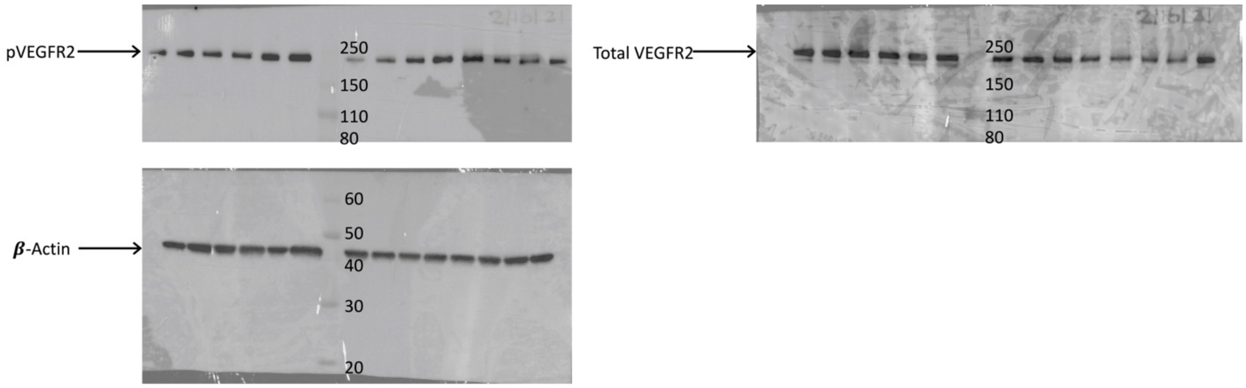

Supplementary Information File 6

(Corresponds to Supplemental Figure 5A)

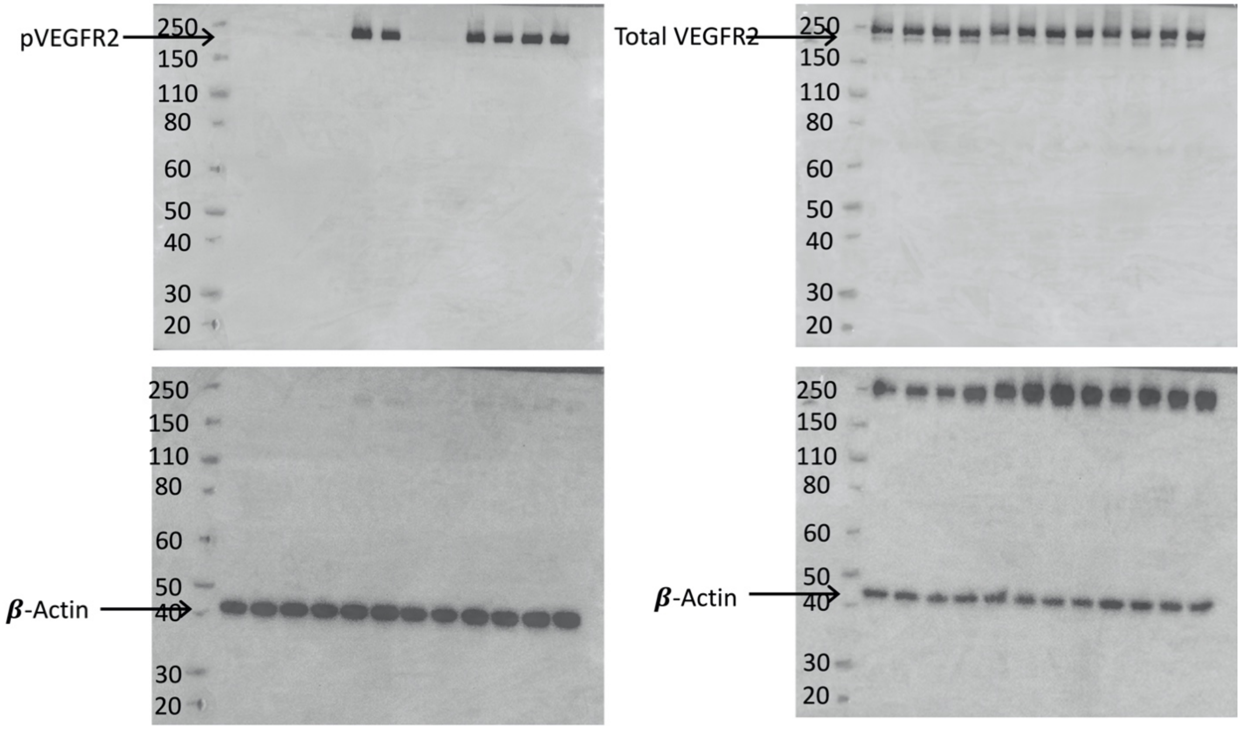

Supplement: Supplementary file 1 — Supplementary Information Files. [file 41598_2021_91127_MOESM1_ESM.pdf]
