# Supplementary material for: Investigation of the mechanisms of VEGF-mediated compensatory lung growth: the role of the VEGF heparin-binding domain
Source: Sci Rep. 2021 Jun 4;11:11827. doi: 10.1038/s41598-021-91127-0 (PMC8178332; doi:10.1038/s41598-021-91127-0)
Supplement: Supplementary file 2 — Supplementary Figures. [file 41598_2021_91127_MOESM2_ESM.pdf]

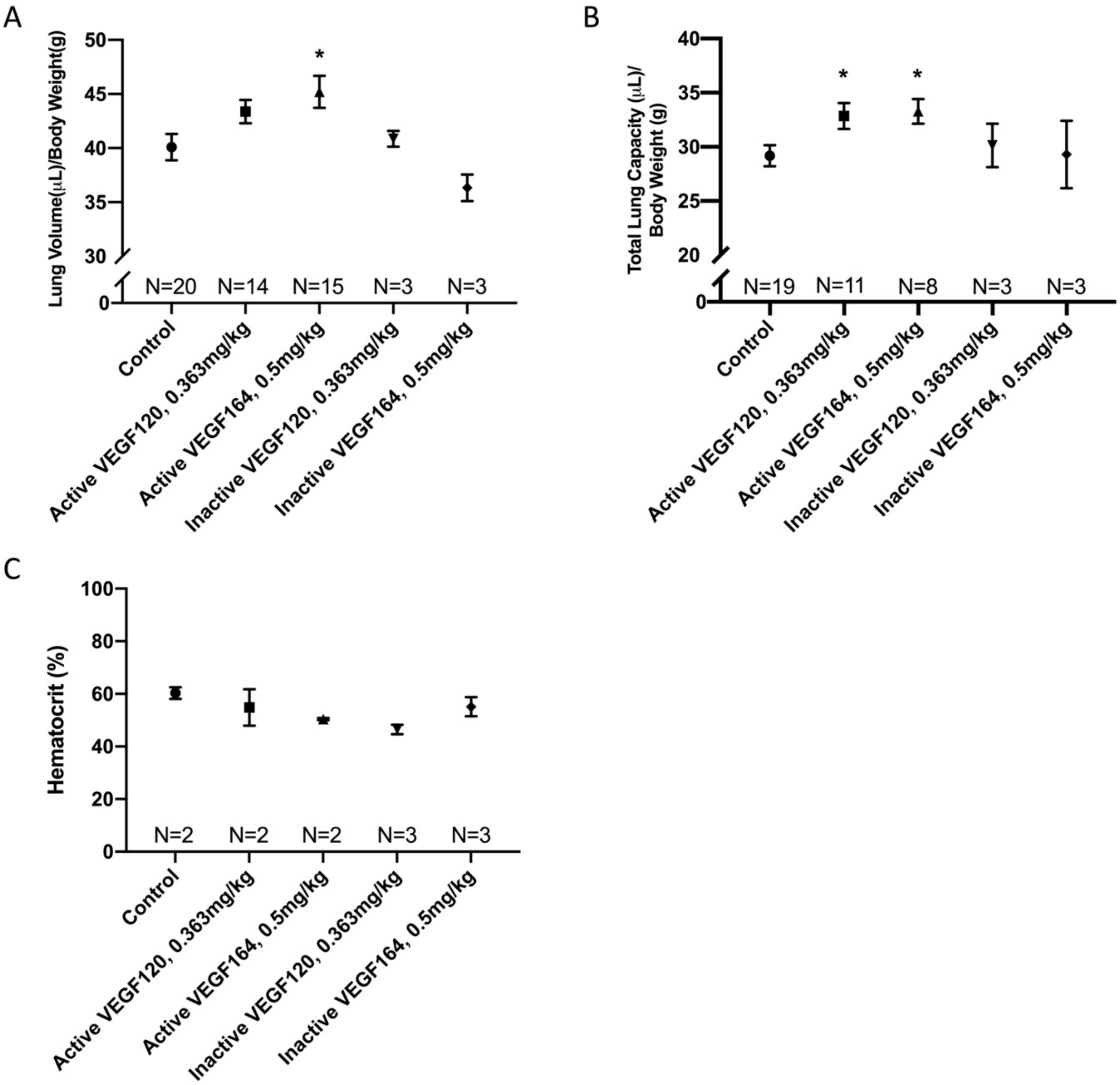

Supplemental Figure 1. Specificity of VEGF treatment

Lung growth stimulated by VEGF120 or VEGF164 treatment over saline control was specific to receiving active protein, as inactive (rendered by boiling) VEGF120 or VEGF164 at the same dose was inert, as demonstrated by lung volume by water displacement **(A)** or total lung capacity **(B)**. Two mice in each of the original groups (Control, Active VEGFs) were added to the respective groups shown in Figure 1, in addition to three mice in each of the inactive VEGF treatment groups. No significant changes were observed in hematocrit **(C).**

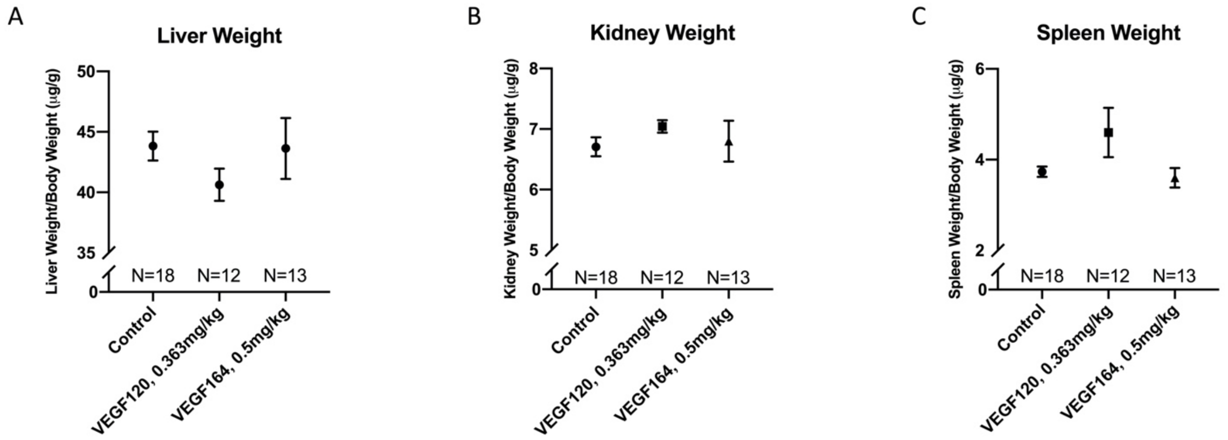

Supplemental Figure 2. Organ weights of mice treated with VEGF
Unlike lung volume, the weights of other, non-regenerating organs, liver **(A)**, right kidney **(B)**, or spleen **(C)**, were not significantly affected by either VEGF120 or VEGF164 treatment. Statistical analysis of organ weights was performed by ANOVA with Holm-Sidak correction for multiple comparisons. Results are expressed as mean ± SE.

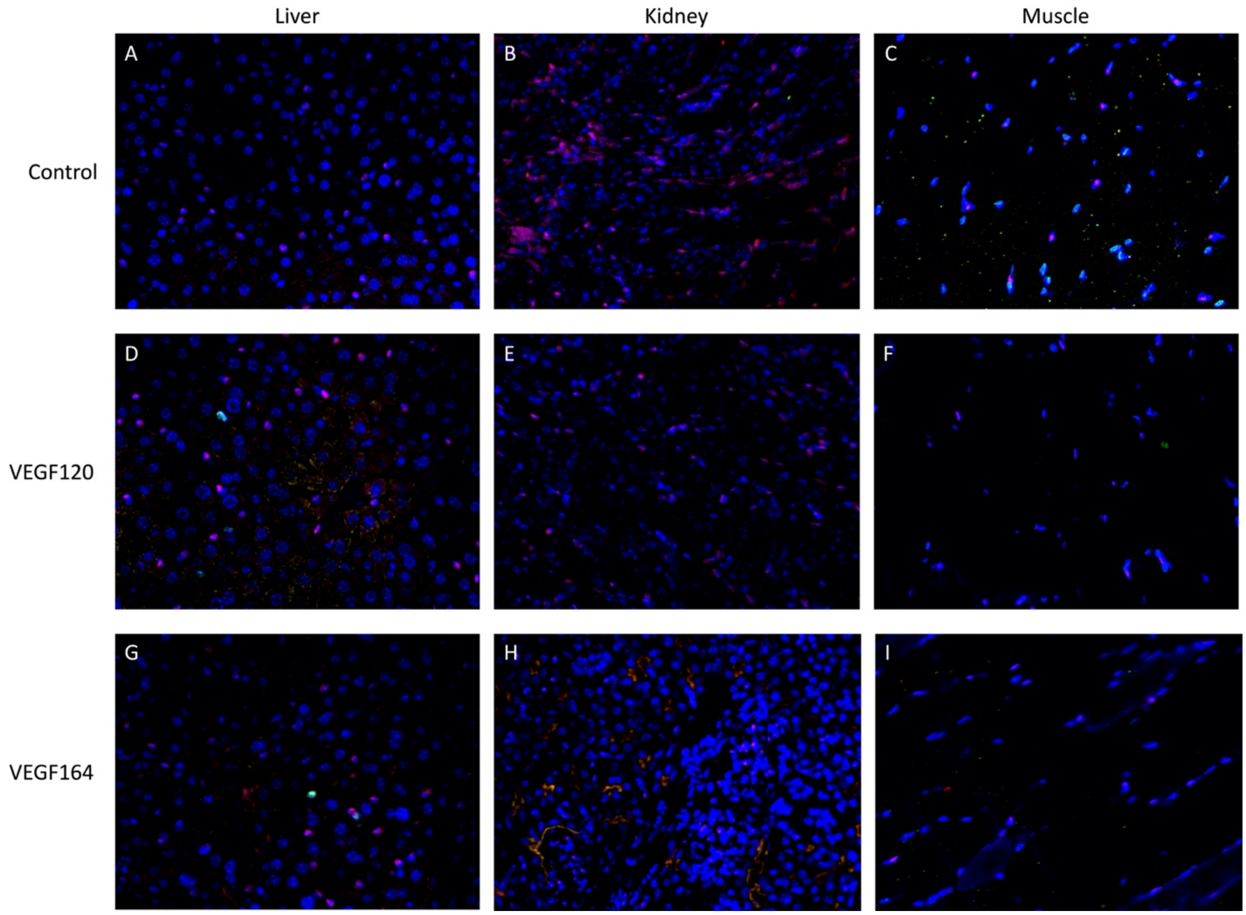

Supplemental Figure 3. Endothelial Cell Proliferation in Non-proliferative Organs. Representative micrographs at 200X magnification of immunofluorescence-stained tissue with Ki-67 (green) and ERG (red) with nuclear DAPI counterstain (blue) demonstrated no substantial endothelial cell proliferation in non-regenerating tissues, liver **(A, D, G)**, kidney **(B, E, H)**, or gastrocnemius muscle **(C, F, I)**. Neither VEGF120 treatment **(D, E, F)** nor VEGF164 treatment **(G, H, I)** increased endothelial cell proliferation in non-regenerative organs over saline control **(A, B, C)**.

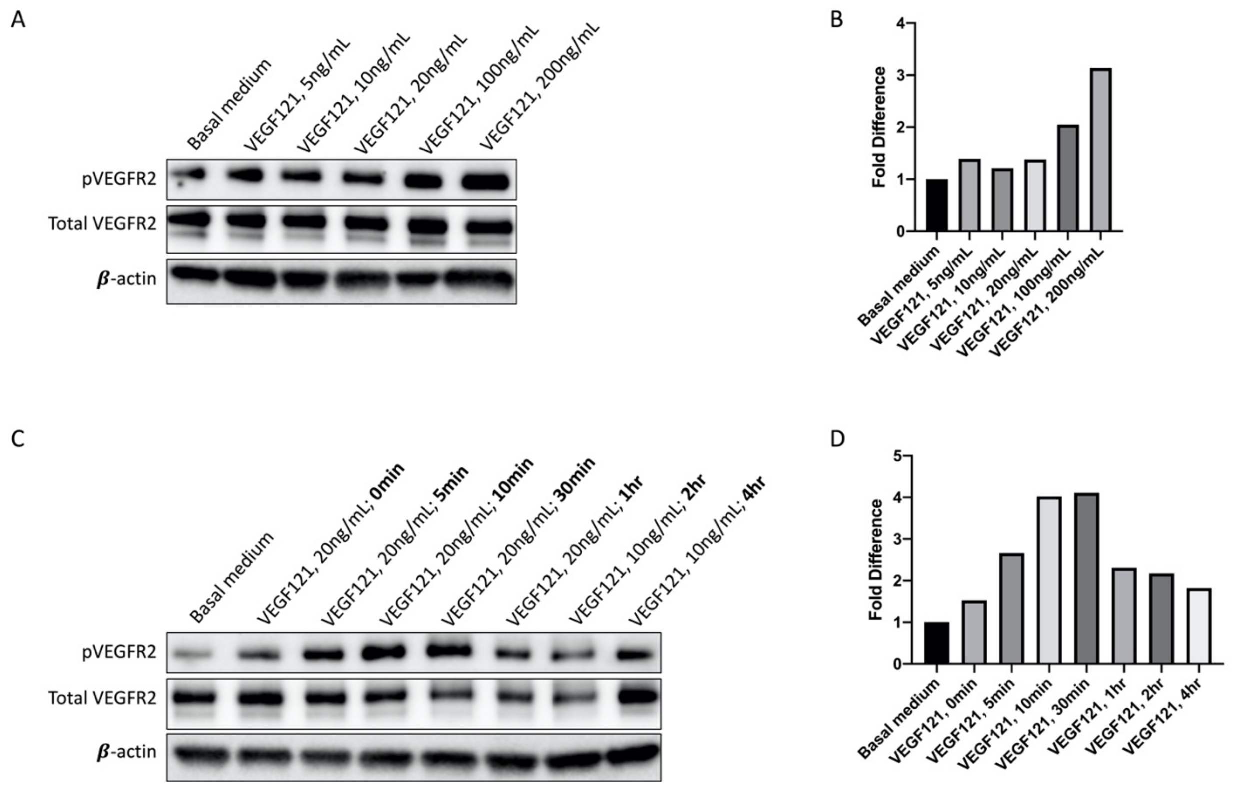

Supplemental Figure 4. *In vitro* VEGFR2 Activation with Extended Dose Response and Timed Induction

VEGF activation of VEGFR2 in human microvascular lung endothelial cells (HMVEC-L) was re-evaluated with an extended dosing range and time intervals for induction. VEGF121 treatment induced a dose-dependent increase in VEGFR2 activation (phosphorylated/total VEGFR2) up to 200ng/mL **(A/B)**. [Full-length uncropped blots are provided in Supplementary Information File 5).] Extended time intervals allowed for induction demonstrated that VEGF121 elicited maximum receptor receptor activation by 10 minutes, sustained this effect for up to 30 minutes, followed by decrease in receptor activation (phosphorylated/total VEGFR2) at longer time intervals **(C/D)**. [Full-length uncropped blots are provided in Supplementary Information File 5).] The ***β***-actin loading control from each membrane is displayed under the image of the primary antibodies of interest. Grouped blots were performed from the same gel with phosphorylated antibody stripped for reprobing with total antibody.

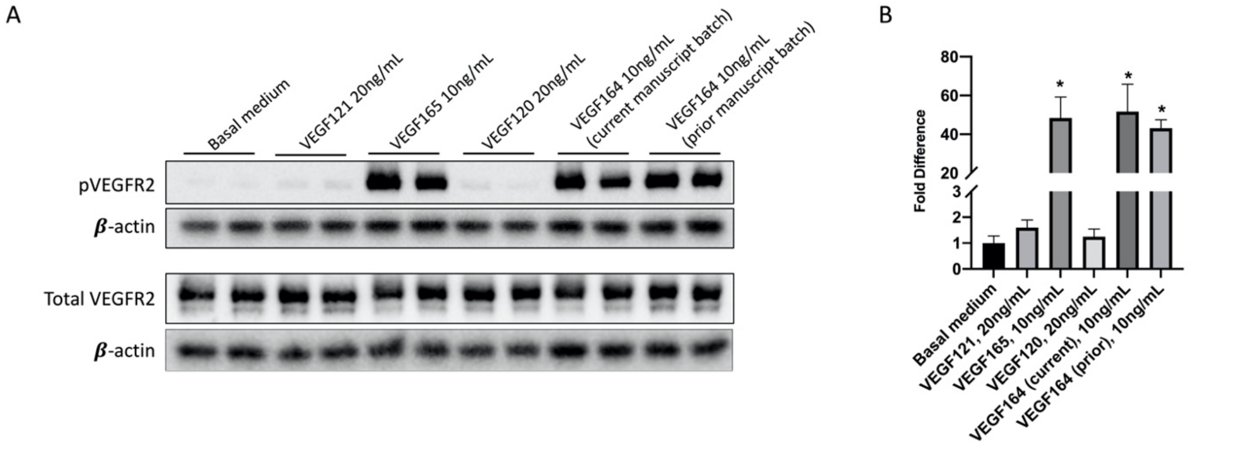

Supplemental Figure 5. *In vitro* VEGFR2 Activation with Multiple Isoforms

VEGF activation of VEGFR2 in human microvascular lung endothelial cells (HMVEC-L) was evaluated using multiple exogenous VEGF isoforms and across species, human and mouse, to encompass assessment of proteins used for *in vivo* experiments. Both VEGF121 (human isoform lacking heparin-binding domain [HBD]) and VEGF120 (mouse isoform lacking HBD) had markedly reduced potency compared to human (VEGF165) and mouse (VEGF164) isoforms containing the HBD, despite treatment at higher concentration. [Full-length uncropped blots are provided in Supplementary Information File 6.] The $\boldsymbol{\beta}$-actin loading control from each membrane is displayed under the image of the primary antibody of interest. Grouped blots were performed from the same protein lysate simultaneously on separate gels. This blot was repeated three times for quality control and a representative image is displayed. Statistical analysis of protein expression quantification from three independent runs was performed by ANOVA with Holm-Sidak correction for multiple comparisons. Results are expressed as mean ± SE. *P<0.05.

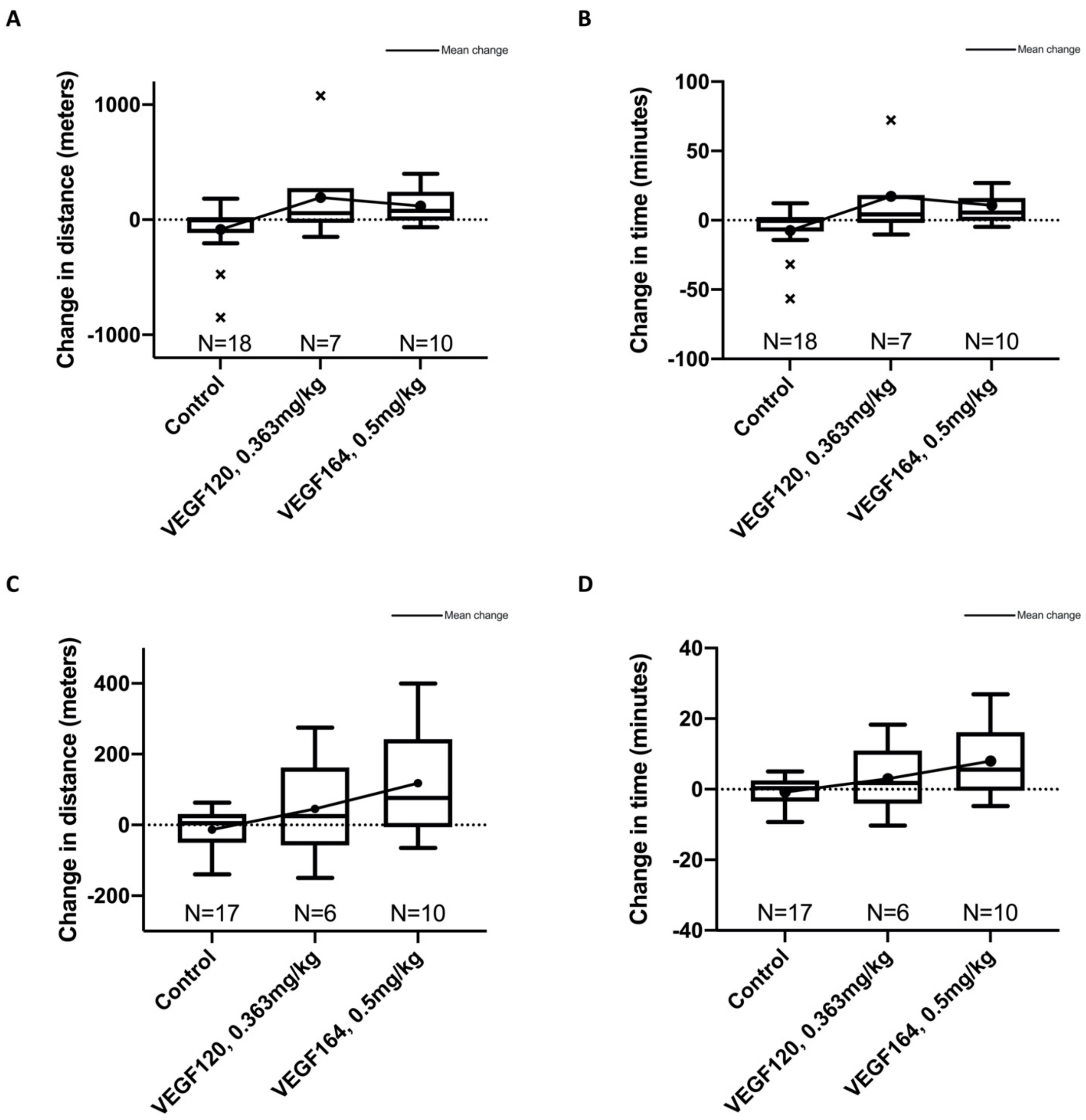

Supplemental Figure 6. Treadmill Exercise Tolerance Testing Analysis

The mean distance and time for the control and VEGF120 groups are heavily influenced by outliers. There were three extreme outliers within the entire cohort of 35 mice, marked by X's on the graphs (**A distance, B time).** Sensitivity analysis performed by eliminating the two most influential outliers (**C, D**), one low-outlier in the control group and one high-outlier in the VEGF120 group, indicated that the outliers rendered the mean values unreliable (compare **A, B** vs. **C, D**). Therefore, to include all the data points and minimize the influence of outliers on the mean, a nonparametric analysis of covariance was performed and reported in **Figure 3**.
