# Supplementary material for: Investigation of the mechanisms of VEGF-mediated compensatory lung growth: the role of the VEGF heparin-binding domain
Source: Sci Rep. 2021 Jun 4;11:11827. doi: 10.1038/s41598-021-91127-0 (PMC8178332; doi:10.1038/s41598-021-91127-0)
Supplement: Supplementary file 3 — Supplementary Method. [file 41598_2021_91127_MOESM3_ESM.docx]

**SUPPLEMENTAL METHODS**

**Statistical Analysis of Exercise Tolerance**

In the treadmill exercise tolerance testing (TETT) experiment, three animals were identified as extreme outliers, which were 3- to 7-fold greater or less in absolute value than the interquartile range (IQR) for change in exercise distance and time. These outliers made comparison of means unreliable and caused the mean change in distance and time to be exceptionally lower for the control group and higher for the VEGF120 group **(Supplemental Figure 6A/B)**. While difficult to pinpoint the exact reason for outliers, these animals may have had intrinsic behavioral or physiologic differences that caused extreme responses to surgery, treatment, or a second round of TETT. Sensitivity to outliers using ordinary ANCOVA was high with *P*=0.12 for differences among groups with outliers present and *P*=0.03 with outliers omitted, as demonstrated by the graphs in **Supplemental Figure 6C/D**, in which the two most extreme outliers have been omitted. As such, in order to retain all the data and minimize the influence of outliers, nonparametric ANCOVA was used to compare treatment groups.[^48^](#_ENREF_48) This method requires that the ranks of the outcome first be regressed on the ranks of the covariate (baseline outcome). Next, the Mantel-Haenszel mean score statistic is used to compare the mean values of residuals across treatment groups. Results for TETT are reported as median and interquartile range (IQR) with *P* value from nonparametric ANCOVA.

**Total Lung Capacity Measurement Using a Flexivent System**

Total lung capacity was measured using a Flexivent system per manufacturer instructions. Briefly, the subject is ventilated with 100% oxygen for five minutes using a closed system to displace other gases from the subject’s lung. Continuing in this closed system, the Flexivent valves then close and ventilation is stopped for an additional five minutes of degassing. All the oxygen present in the subject’s lungs will be absorbed into the blood during this period, and the lungs will collapse. In addition, with the lack of ventilation and effective tracheal occlusion during this degassing period, the animal is euthanized as the heart stops. With lack of perfusion, the exchange of CO2 in the lungs is also halted. Following complete degassing, the Flexivent piston pushes air into the subject’s lung at a constant flow rate until a pressure of 35 cmH_2_O is reached. This is performed mechanically by the machine and should be replicable with any Flexivent system calibrated to perform this maneuver. The Flexivent piston then draws air back out of the lungs until a pressure of -10 cmH_2_O is reached and repeats the inflation/deflation maneuver for a total of 3 complete runs to measure the total lung capacity, which is in the mouse, is the total volume able to be achieved at a certain maximum pressure and is constant across subjects.
